# mTOR complexes differentially orchestrates eosinophil development in allergy

**DOI:** 10.1038/s41598-018-25358-z

**Published:** 2018-05-02

**Authors:** Chen Zhu, Lixia Xia, Fei Li, Lingren Zhou, Qingyu Weng, Zhouyang Li, Yinfang Wu, Yuanyuan Mao, Chao Zhang, Yanping Wu, Miao Li, Songmin Ying, Zhihua Chen, Huahao Shen, Wen Li

**Affiliations:** 1 Department of Respiratory and Critical Care Medicine, The Second Affiliated Hospital, Zhejiang University School of Medicine, Hangzhou, Zhejiang 310009 China; 2 Department of Respiratory Medicine, Ningbo No. 2 Hospital, Ningbo, Zhejiang 315010 China; 3 Department of Pharmacology, Zhejiang University School of Medicine, Hangzhou, Zhejiang 310058 China

## Abstract

Eosinophil infiltration is considered a hallmark in allergic airway inflammation, and the blockade of eosinophil differentiation may be an effective approach for treating eosinophil-related disorders. Mammalian target of rapamycin (mTOR) is a vital modulator in cell growth control and related diseases, and we have recently demonstrated that rapamycin can suppress eosinophil differentiation in allergic airway inflammation. Considering its critical role in haematopoiesis, we further investigated the role of mTOR in eosinophil differentiation in the context of asthmatic pathogenesis. Intriguingly, the inhibition of mTOR, either by genetic deletion or by another pharmacological inhibitor torin-1, accelerated the eosinophil development in the presence of IL-5. However, this was not observed to have any considerable effect on eosinophil apoptosis. The effect of mTOR in eosinophil differentiation was mediated by Erk signalling. Moreover, myeloid specific knockout of mTOR or Rheb further augmented allergic airway inflammation in mice after allergen exposure. Ablation of mTOR in myeloid cells also resulted in an increased number of eosinophil lineage-committed progenitors (Eops) in allergic mice. Collectively, our data uncovered the differential effects of mTOR in the regulation of eosinophil development, likely due to the distinct functions of mTOR complex 1 or 2, which thus exerts a pivotal implication in eosinophil-associated diseases.

## Introduction

Allergic asthma is a chronic airway inflammation characterized by reversible airflow limitation, airway hyperresponsiveness, and progressive infiltration of inflammatory cells. Among all inflammatory cells, eosinophils and eosinophil-secreted cytokines play a dominant role in asthmatic pathogenesis^1,2^. Recently, eosinophils have been shown to regulate Th2 response, and appear to correlate with the severity of asthma^3,4^. In asthmatic patients, eosinophils first develop in bone marrow from progenitor cells^5–7^. When exposed to allergens, eosinophil lineage-committed precursors exhibit elevated differentiative and migratory capabilities into peripheral blood and lung tissue under the presence of eotaxins^8^.

Eosinophils, neutrophils, and basophils are the main constitution of granulocytes, and share a progenitor called granulocyte/monocyte progenitor (GMP). GMPs can differentiate to mature eosinophils during a process called intermediate eosinophil lineage-committed progenitors (Eops) under the control of several transcription factors, including PU.1, C/EBP^6,9^, and GATA-1, the deficiency of which failed to induce eosinophilia^10^. Eops begin to express IL-5 receptor, therefore having the potential to differentiate toward eosinophils in response to IL-5^11^. An increasing amount of evidence suggests that eosinophils plays a crucial regulatory role in a wide range of diseases, including for instance allergic diseases^3,4,12^, parasite infections, autoimmune diseases, and even cancer^13^.

Mammalian target of rapamycin (mTOR) is a serine/threonine protein kinase known for its varieties of regulatory roles in cell survival, cell differentiation, protein synthesis, and glycolysis^14,15^. mTOR interacts with various proteins to form two distinct components, named mTOR complex 1 (mTORC1) and mTOR complex 2 (mTORC2). mTORC1 contains a Raptor sensitive to rapamycin, whereas mTORC2 is insensitive to rapamycin due to the rapamycin insensitive domain Rictor.

mTOR is essential in the hematopoietic system. Kalaitzidis *et al*. have reported that mTORC1 is required for hematopoietic stem cell (HSC) function after bone marrow transplantation in murine models, and that mice receiving Raptor null HSCs may not survive post lethally irradiated^16^. Likewise, research has revealed that S6K1, a downstream of mTORC1, plays a crucial role in early adipocyte differentiation. However, the ablation of S6K1 or treatment of rapamycin can suppress this process^17^. mTOR also participates in CD8^+^ T cell differentiation. Interestingly, CD8^+^ T cell effector response is mainly influenced by mTORC1, whereas memory generation is largely affected by mTORC2^18^. Macrophage polarization is selectively regulated by the mTORC1-Akt-TSC1 loop^19^, and its regulatory role may be reversed when facing acute activation or chronic activation of mTORC1. In eosinophil-related research, the loss of mTOR can lead to autophagy activation, which has been observed in the peripheral blood eosinophils of severe asthma patients^20,21^. Therefore, this indicates that mTOR may contribute to asthmatic pathogenesis through regulation of eosinophil development or function.

It has been reported that rapamycin administration can attenuate OVA-induced allergic airway inflammation^22,23^. Interestingly, the role of rapamycin remains paradoxical in asthma models induced by the house dust mite^24^. We have demonstrated that rapamycin can suppress eosinophil differentiation, therefore reducing airway inflammation^23^. To demonstrate the effect of mTOR in eosinophil development, we further established a murine strain with specific deletion of mTOR in myeloid cells. However, by using these mTOR specific knockout mice, we discovered more aggressive eosinophil development, resulting in elevated levels of eosinophil infiltration in allergies. Therefore, these differential effects of mTOR in the regulation of eosinophil differentiation may be caused by the distinct functions of mTORC1 and mTORC2.

## Materials and Methods

### Mice

Mtor^flox/flox^ mice on a background of C57BL/6 were purchased from Jackson Lab, and LysM^Cre^ mice on a background of C57BL/6 were a generous gift from Dr. Gensheng Feng (University of California at San Diego, CA, USA). All mice were housed in the Laboratory animal center of Zhejiang University, Hangzhou, China. Age-matched and gender-matched mice were used in experiments according to Zhejiang University Medical Laboratory Animal Care and Use Committee.

All experiments involving animals were strictly performed in accordance with the stipulations and protocols approved by Ethics Committee for Animal Studies at Zhejiang University. Sample size was chosen based on similar experiments in published articles. Animals were chosen randomly for each group; single blind was used in BALF counting. The primers used for genotyping were as follows: Mtor: forward, 5′-TTATGTTTGATAATTGCAGTTTTGGCTAGCAGT-3′; reverse, 5′ TTTAGGACTCCTTCTGTGACATACATTTCCT-3′; LysM^Cre^: mutant primer, 5′- CCCAGAAATGCCAGATTACG-3′; common primer, 5′-CTTGGGCTGCCAGAATTTCTC-3′; wildtype primer, 5′-TTACAGTCGGCCAGGCTGAC-3′.

### Isolation and culture of murine bone-marrow-derived-eosinophils (BMDEs)

The protocols, isolation, and culture of mouse eosinophils were fully described elsewhere^25–28^. Generally, bone-marrow derived non-adherent mononuclear cells (NAMNCs) were seeded at 1 × 10^6^/ml in IMDM completed medium containing IMDM (Iscove’s modified Dulbecco’s medium; Invitrogen, Waltham, MA, USA) with 20% FBS (Gibco; origin from Australia), 100 IU/ml penicillin and 10 mg/ml streptomycin, 2mM L-glutamine, 1 × non-essential amino acids (Sigma-Aldrich, St. Louis, MO, USA), 1 mM sodium pyruvate (Sigma-Aldrich) and 0.006‰ β-mercaptoethanol (Sigma-Aldrich). 100 ng/ml rmFlt-3L (Peprotech, Rocky Hill, NJ, USA), and 100 ng/ml rmSCF (Peprotech) were supplemented from day 0 to day 4. Medium was replaced on day 4 and day 8, containing 10 ng/ml rmIL-5 (R&D Systems, Minneapolis, MN, USA), but without rmFlt-3L and rmSCF. Most experiments were performed in day 8 to day 10. Torin-1 (Tocris Biosciences) and U0126 (Selleck) were treated from day 4 when the medium was replaced. Cells were harvested and detected using PE-conjugated anti-SiglecF, and apoptotic levels were analyzed using AnnexinV-FITC and 7-AAD. Cells were lysed and detected by western blotting with p-S6 (Cell signaling technology, Denver, MA, USA), LC3B, Erk1/2, p-Erk1/2 and β-actin and analyzed Mbp and Gata-1 mRNA levels using quantitative real-time PCR.

### Mtor *ex vivo* deletion in bone marrow cells by adenovirus transfection

NAMNCs from C57BL/6 or Mtor^flox/flox^ mice were collected as previously described, then transfected with Ade-Cre-GFP and Ade-GFP at a multiplicity of infection of 50 for 6 hours on the fourth day. All further procedures were as previously described^25–27^.

### Colony-forming Units

A total of 2 × 10^5^ NAMNCs were cultured in IMDM completed medium supplemented with 0.9% methylcellulose (Stem Cell) and 10 ng/ml rmIL-5 in 3.5 cm dishes (Corning, NY, USA) at 37 °C and 5% CO_2_ as described elsewhere^26,27^. Colonies (of more than 40 cells) were calculated at day 10 by using an inverted microscope. Eos-CFU was picked and classified by flow cytometric assay and histological evidence.

### Flow cytometry

To identify eosinophil from other cells in bone marrow, cells were stained with PE-conjugated SiglecF (BD Pharmigen), or together with PE-Cy7-conjugated anti-F4/80. Eosinophils were described as SiglecF^+^F4/80^+^ or SiglecF^+^SSC^hi^ ^27^. Apoptosis cells were detected by using Annexin-V and 7-AAD kits (Multi Sciences) according to manufacturer protocols.

For the identification of eosinophil-associated hematopoietic progenitors from LSK (Lineage^−^ c-kit^−^ Scal-1^+^ CD34^+^) to Eops (Lineage^−^ CD34^+^ CD16/32^hi^ c-kit^low^ IL-5Ra^+^), antibodies were selected as follows^26,27^: Biotin-conjugated Lineage cocktail (CD4, RM4-5; CD8a, 53-6.7; CD11b, M1/70; CD45R/B220, RA3-6B2; Gr-1, RB6-8C5; Ter-119, Biolegend), streptavindin-APC-Cy7 (BD Pharmigen), FITC-conjugated anti- IL-5Ra, APC-conjugated anti-c-kit (2B8, eBiosciences), PE-Cy7-conjugated anti-Sca-1 (eBiosciences), Percp-Cy5.5-conjugated anti-CD16/32 (eBiosciences), and Alexa Fluor 700-conjugated anti-CD34 (RAM34; eBioscience). Dead cells were excluded as DAPI^+^ cells.

For T helper cell subset detection, lung tissues were digested by collagenase I (C0130, Sigma Aldrich), then stained by APC-eFluor 780-conjugated anti-CD3 (17A2, eBioscience), and PE-Cy7-conjugated anti-CD8 (BD Pharmigen). After fixation and permeabilization, BV510-conjugated anti-CD25 (PC61, Biolegend), FITC-conjugated anti-IFN-γ (XMG1.2, Biolegend), PE-conjugated anti-IL-13 (eBio13A, eBioscience), APC-conjugated anti-IL-17a (eBio17B7, eBioscience), and Pacific Blue-conjugated anti-Foxp3 (MF-14, Biolegend) were marked. The distinguishing of Th1 (IFN-γ^+^), Th2 (IL-13^+^), Th17 (IL-17a^+^), and Treg (CD25^+^Foxp3^+^) was performed on the basis of T helper cells (CD3^+^CD8^−^).

Stained cells were analysed by FC500 or LSRFortessa. Data were re-analyzed using FlowJo software (Treestar Inc.).

### Ovalbumin-induced allergic airway inflammation mouse model

Mice were sensitized on day 0 and day 14 with OVA mixed with aluminium adjuvants by intraperitoneal injection (i.p.), then challenged with 1.5% OVA or saline for 45 minutes at day 24 to 26, and parameters were analysed 24 hours after final OVA challenge^4,23,26,27^. Mice were euthanized after being anesthetized by 1.5% amobarbital, and BALF, blood, lung tissues, and bone marrow cells were collected for further analyses. Eosinophils were detected in bone marrow and peripheral blood using flow cytometry was counted using BALF meeting histological criteria. Lung tissues were fixed and stained with H&E and PAS, slides were visualized by Olympus BX51 microscope (4/0.3 NA objective), and equipped with an Olympus DP70 digital camera.

The inflammation score was graded by two independent blinded investigators. The concentration of peribronchial and perivascular inflammation was assessed according to a subjective scale from 0 to 3 as fully described elsewhere^29^. A grade of 0 was classified as no inflammation; a grade of 1 represented infrequent cuffing with inflammatory cells; a grade of 2 represented the numbers of bronchi or vessels embraced by a thin layer of inflammatory cells; a grade of 3 symbolized that most bronchi or vessels were surrounded by a dense layer of inflammation. The concentration of IL-4 and IL-13 was detected using Elisa kits from R&D systems.

### Purification of RNA and quantitative real-time PCR

Cells or tissues were lysed by Trizol reagent (Takara, Kusatsu, Shiga, Japan) according to manufacturer’s instruction. Primers for PCR were synthesized by Shanghai Bioengineering (Shanghai, China). cDNA was reverse transcribed to cDNA using a PrimeScript TM RT-PCR kit (Takara) from total RNA, then subjected to quantitative real-time PCR with SYBR Primix Ex TaqTM (Takara). QPCR was performed on a 7500 Real-time PCR system (Applied Biosystems, Carlsbad, CA, USA). The primers used are as follows: IL-4: forward, 5′-GGTCTCAACCCCCAGCTAGT-3′; reverse, 5′-GCCGATGATCTCTCTCAAGTGAT-3′; IL-13: forward, 5′-CAGCCTCCCCGATACCAAAAT-3′; reverse, 5′-GCGAAACAGTTGCTTTGTGTAG-3′; Gata-1: forward, 5′-TATGGCAAGACGGCACTCTAC-3′; reverse, 5′-GGTGTCCAAGAACGTGTTGTT-3′; Mbp: forward, 5′-GCAAACGCTTTCGATGGGTTG-3′; reverse, 5′-ACACAGTGAGATAGACGCCAG-3′; β-actin: forward, 5′-AGAGGGAAATCGTGCGRGAC-3′; reverse, 5′-CAATAGTGACCTGGCCGT-3′. The delta-delta CT method analyzed mRNA expression, and the relative fold was normalized to β-actin.

## Results

### Torin-1 or genetic mTOR knockdown enhances eosinophil differentiation

**Figure 1:**
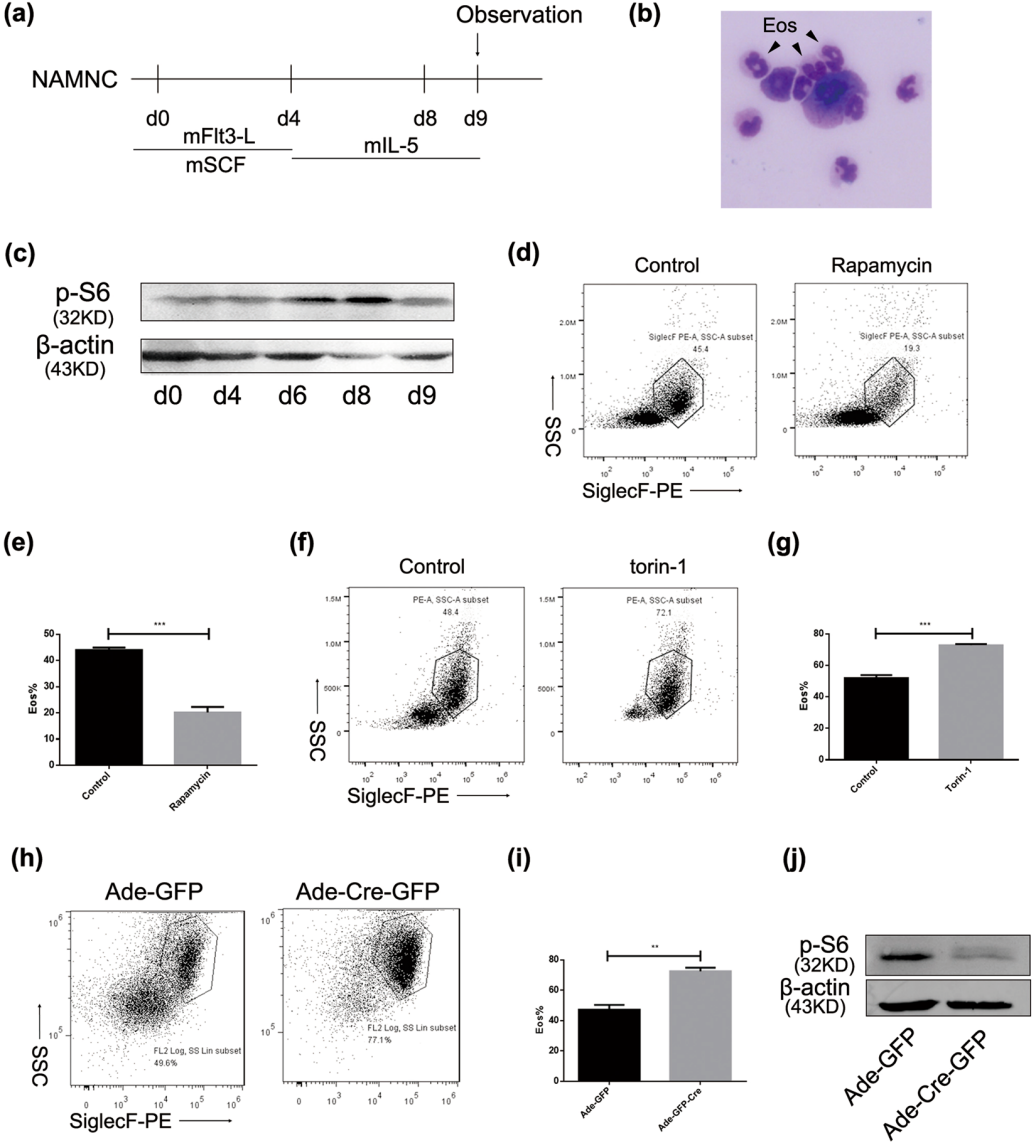
Torin-1 administration and genetic deletion of mTOR contributes to elevated differentiation of eosinophils. (**a**) Bone-marrow derived eosinophils (BMDE) during culture processes were harvested and mTOR (displayed as p-S6) was detected by western blot assay. (**b**) BMDE culture *in vitro*. Non-adherent mononuclear cells from bone marrow were obtained and differentiation was conducted with rmFlt-3L, rmSCF, and rmIL-5. Western Blot are performed according to auto-exposure and the resolution of 169 μm. Given that the highest level of p-S6 expression was on day 8, torin-1 or rapamycin was treated on day 8. (**c**) Blots were cropped from the same gel.) Mature eosinophils underwent Wrights-Giemsa staining after being cytospined. Images were acquired from an Olympus BX51 microscope (x4/0.3 NA objective with Olympus DP70 digital camera) for acquisition of images. (**d**,**e**) Percentage of eosinophils after rapamycin treatment (50 nM) was detected by flow cytometry (d) and statistically analysed as means ± SEM using Student’s T-test (e). (**f**,**g**) Percentage (g) of eosinophils after torin-1 administration (250 nM) was detected by flow cytometry and statistically analysed as means ± SEM (g). (**h**,**i**) Adenovirus with Cre recombinase was harvested from HEK293T cells and the titer measurement was undertaken. Bone-marrow-derived cells were infected with adenoviral administration 6 hours without serum. Percentage (h) of eosinophils was detected by flow cytometry and statistically analysed as means ± SEM (i). All the results above were triplicated by three independent experiments. Efficiency of adenoviral knockdown (**j**) Blots were according to different gels.) was detected on day 9. *P < 0.05, **P < 0.01, ***P < 0.001.

Firstly, non-adherent mononuclear cells were harvested from mice bone marrow and cultured in IMDM completed medium containing FLT-3 ligand (rmFlt-3L, 100 ng/ml) with stem cell factor (rmSCF, 100 ng/ml) or murine interleukin-5 (rmIL-5, 10 ng/ml) (Fig. 1a). Developed eosinophils consisted of doughnut-like granules, and morphologic characteristics could be precisely observed after Wright-Giemsa staining (Fig. 1b). To investigate the function of mTOR in the process of eosinophil development, we first analyzed mTOR activity as revealed by phospho-S6 (pS6) in the process of eosinophil development (Fig. 1c). pS6 expression was increased upon IL-5 stimulation, and peaked on day 4. Accordingly, we treated BMDE with torin-1, a potent ATP-competitive mTOR inhibitor on day 4. Compared to rapamycin, torin-1 had the ability to inhibit both mTORC1 and mTORC2, and revealed at least 100-fold selectivity^30,31^. SiglecF, also called Siglec-5, is the distinctive surface marker of eosinophils. Accordingly, we defined eosinophils as SiglecF^+^F4/80^+^ in flow cytometry analysis or SiglecF^+^SSC^hi^ due to their complex intercellular components. Rapamycin administration resulted in diminished eosinophil differentiation (Fig. 1d and e), as previously reported^23^. However, torin-1 administration led to enhanced levels of eosinophil differentiation in the presence of IL-5 (Fig. 1f and g). To further verify this phenomenon, NAMNCs from Mtor^flox/flox^ mice were treated with adenovirus expressing Cre recombinase, and eosinophil generation was also increased after adenoviral administration (Fig. 1h and i). Western blot analysis confirmed the depletion level of mTOR, as revealed by the levels of p-S6 (Fig. 1j).

### Torin-1 or mTOR deficiency increases eosinophil colony forming units

**Figure 2:**
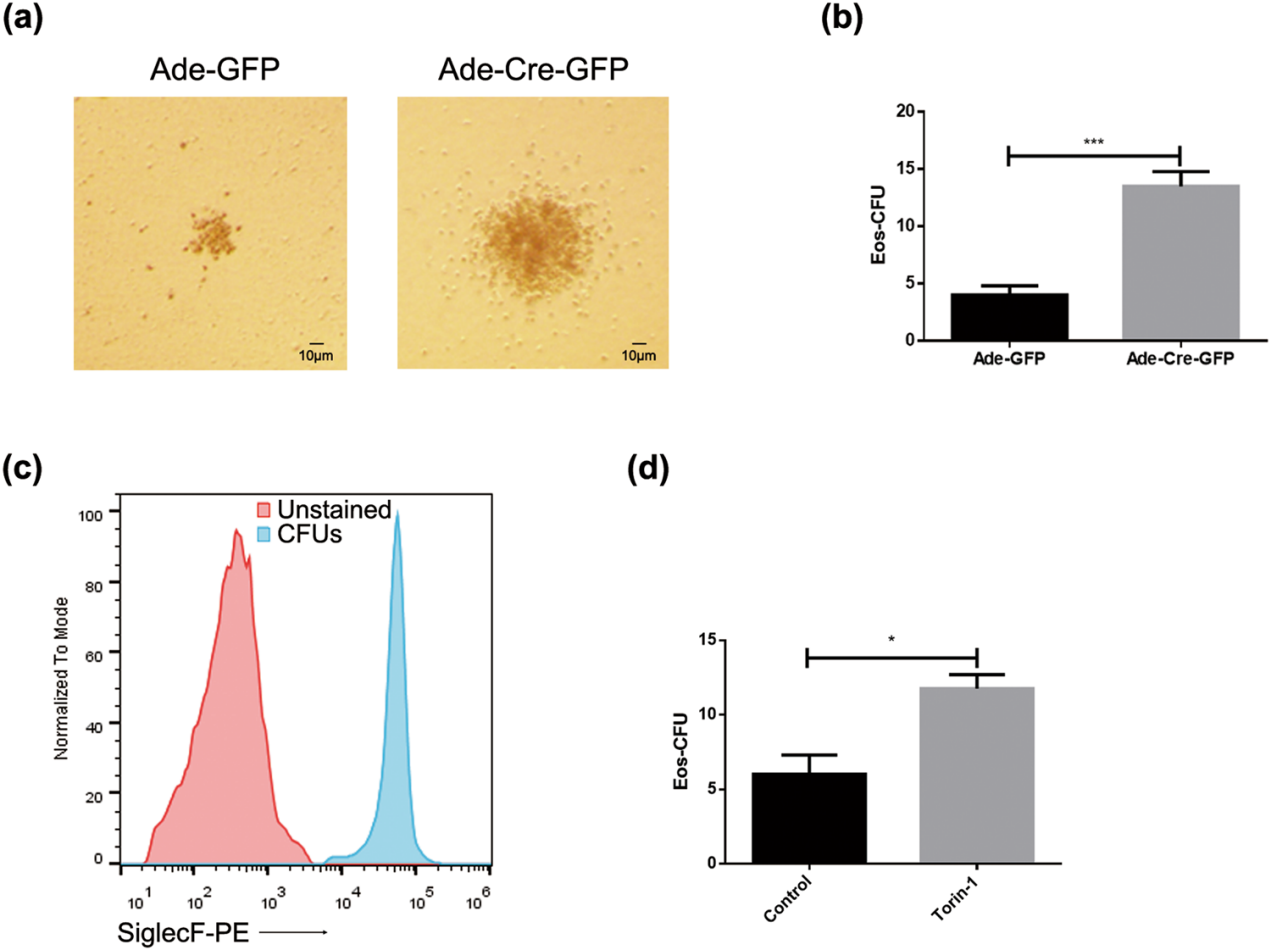
mTOR knockdown promotes IL-5-induced colony forming units. (**a**) 2 × 10^5^ NAMNCs originated from Mtor^flox/flox^ mice were seeded in methylcellulose-based semi-solid medium for colony formation in the presence of IL-5 (10 ng/ml). CFUs were calculated at day 7, and images were captured by microscope. (**b**) CFU numbers after adenoviral Cre recombinase expression. (**c**) CFUs were picked and washed by PBS, then stained with PE-conjugated SiglecF, representative histograms are shown. (**d**) Torin-1 (250 nM) was added in the medium before being seeded, and the number of CFUs were counted on day 7. All the results above were triplicated by three independent experiments and were analysed by Student’s T-test as means ± SEM. *P < 0.05, ***P < 0.001.

To further verify the role of mTOR in IL-5 induced eosinophil development, a colony forming unit (CFU) assay was executed. In agreement with the results shown in Fig. 1, the sizes of CFUs after mTOR deletion were larger when compared with those of the control group (Fig. 2a) and the number of CFUs was also significantly increased in the mTOR deficiency cells (Fig. 2b). Flow cytometry analysis revealed that most of the CFUs were SiglecF positive, suggesting those colonies were indeed eosinophils (Fig. 2c). Similarly, torin-1 treatment also resulted in larger sized and increased number of CFUs (Fig. 2d). Interestingly, the number of G-SCF-induced CFUs was significantly decreased (Fig. S1A) in the mTOR knockdown cells, whereas no effect was observed in M-CSF-induced colony forming (Fig. S1B). Therefore, the data above suggested that mTOR selectively suppressed eosinophil differentiation.

### Myeloid specific knockdown of mTOR *in vivo* enhances eosinophil percentage in bone marrow without altering apoptotic level

**Figure 3:**
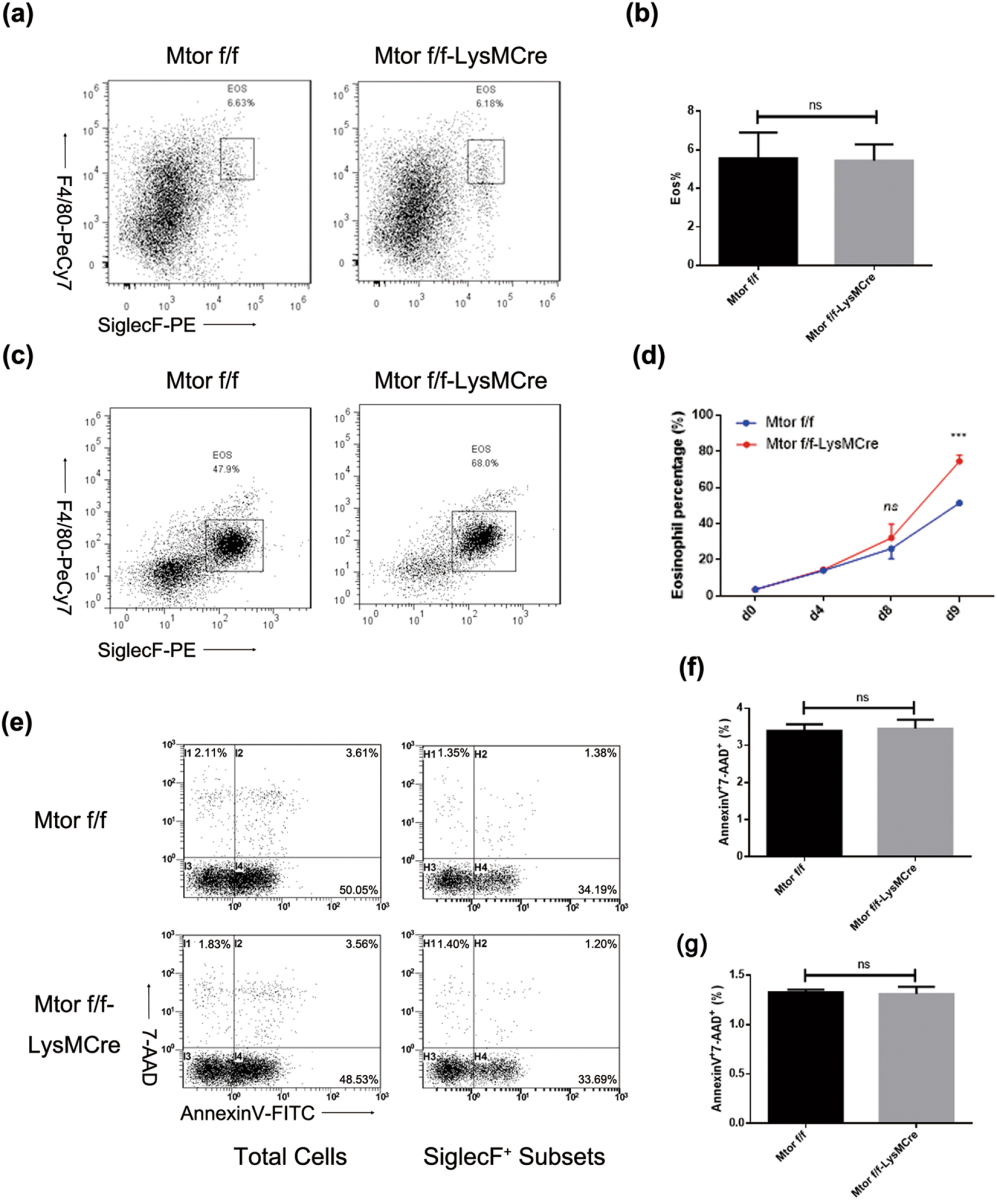
Myeloid specific knockout of mTOR results in elevated levels of eosinophil development without altering apoptotic levels. (**a** and **b**) Bone marrow cells were harvested and stained with PE-conjugated SiglecF and Pe-Cy7-conjugated F4/80 post lysing of red blood cells. Representative dot plots are shown (a) and statistical analysis (b) was conducted as means ± SEM using Student’s T-test. (**c**,**d**) Liquid culture of BMDE with two strains of mice. Representative dot plots are displayed (c) and statistically analysed on day 8 and 9 (d). (**e**–**g**) On day 9, cells were harvested and stained with SiglecF-PE, then stained with AnnexinV-FITC and 7-AAD before flow cytometric analysis. Representative dot plots are shown (e), and analysis of the percentage of apoptotic cells (regarded as AnnexinV^+^7-AAD^+^) were calculated in total cells (f) and eosinophils (SiglecF^+^ subsets, g) All the results above were repeated by three independent experiments. n.s. not significant, *P < 0.05, **P < 0.01, ***P < 0.001.

To further clarify the regulatory role of mTOR in eosinophil differentiation *in vivo*, we established LysM^Cre^ Mtor^flox/flox^ mice whereby Cre recombinase expression was specific expression in myeloid cell lineage. Genomic DNA was extracted from tails to validate the floxed mTOR and Cre expression under the control of the endogenous Lyz2 promotor. Firstly, we analysed the levels of eosinophils in bone marrow without any treatment between these two strains, and discovered that WBC and eosinophil numbers and percentages all remained at the same level in peripheral blood (Figs 3a and b and S2A–C). After culturing, however, the eosinophil percentage remarkably increased in LysM^Cre^ Mtor^flox/flox^ mice (Fig. 3c and d), which is consistent with the *in vitro* culture data shown in Figs 1 and 2. To clarify whether mTOR was involved in apoptosis, eosinophil and total cells from culture dishes were analysed by flow cytometry staining with FITC-conjugated AnnexinV and 7-AAD. The results suggested no difference in apoptotic level after mTOR genetic excision (Fig. 3e–g). To further confirm the role of mTOR in eosinophil apoptosis, we cross-bred LysM^Cre^ Mtor^flox/flox^ mice with IL-5 transgenic (NJ1638) mice, and in those mice, most of the cells in the bone marrow were eosinophils (Fig. S3A). Again, total bone-marrow-derived cells were harvested after splitting red blood cells (Fig. S3B), and no significant apoptosis was discovered in total cells (Fig. S3C) and SiglecF-positive eosinophils (Fig. S3D). Altogether, mTOR specialized deletion in myeloid exerted no effects on the basal levels of eosinophils and their apoptosis, but enhanced the ability of eosinophil development.

### mTOR specific deletion expedites the production of Eops in OVA-induced allergic mice

**Figure 4:**
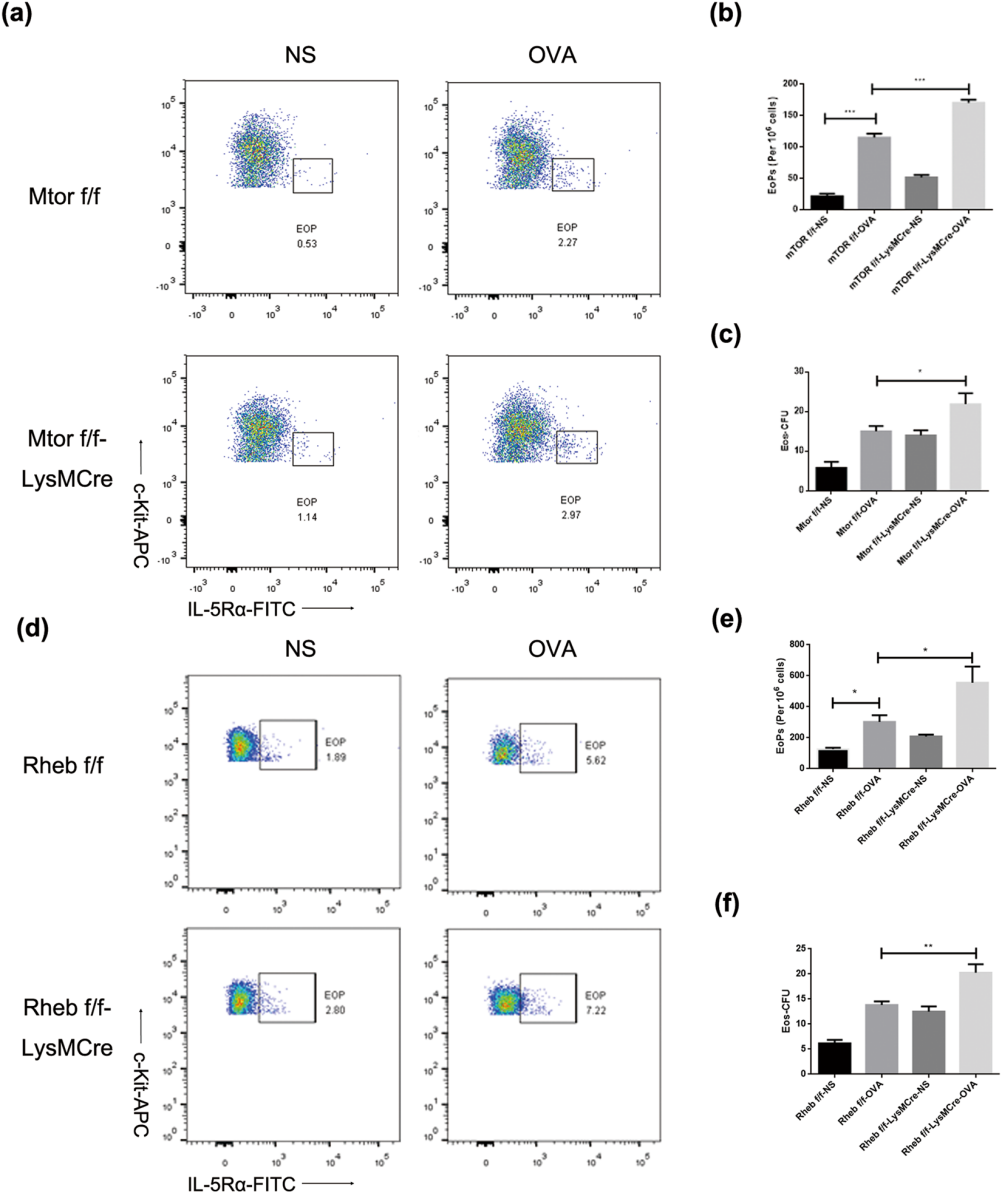
Myeloid specific deletion of mTOR leads to increased level of Eops. (**a**) Eops were identified as c-Kit^+^IL-5Ra^+^ cells in alive Lineage^−^CD34^+^CD16/32^+^ subsets. Representative contour plots of staining were shown using allergic LysM^Cre^ Mtor^flox/flox^ mice (n = 5 per group). (**b**) Number of Eops per 10^6^ cells were statistically calculated as means ± SEM. (**c**) Bone marrows of allergic mice were harvested and colony formation was performed to verify Eops number. (**d**) LysM^Cre^ Rheb^flox/flox^ mice were used to establish OVA-induced asthma model, Eops numbers were detected as previously described. (**e**) Statistical results of Eops in allergic Rheb knockout mice in per 10^6^ cells. (**f**) CFUs of allergic Rheb genetic deletion mice. Results were analysed as means ± SEM. All the results above were repeated by three independent experiments. *P < 0.05, **P < 0.01, ***P < 0.001.

To explore the alternation in progenitors after mTOR knockdown in eosinophil development, we analyzed the lineage of eosinophilopoiesis, including LSKs, CMPs, GMPs (Lineage^−^ c-Kit^+^ CD16/32^hi^ CD34^+^), and Eops (Lineage^−^ CD34^+^ Sca-1^−^ c-Kit^+^ IL-5Ra^+^) using FACS, and the stain strategy was described in detail elsewhere^26,27^. According to these results, the number of Eops was dramatically increased in OVA/LysM^Cre^ Mtor^flox/flox^ mice (Fig. 4a and b). However other eosinophil progenitors showed no alternation (Fig. S4A–C). Accordingly, it is possible to imply that, in allergic mice, accelerated eosinophil differentiation after myeloid-specific deletion of mTOR may be attributable to increased number of Eops. For further validation, OVA/LysM^Cre^ Rheb^flox/flox^ mice also displayed an augmented number of Eops (Fig. 4d and e). However, no change in other early-stage precursors was identified (Fig. S4D–F). Colony forming assay was also conducted to demonstrate the over-production of eosinophil progenitors. Allergic mice were sacrificed, and NAMNCs were extracted from bone marrows and then seeded in methylcellulose media afterwards. We discovered an increased amount of eosinophil CFU in OVA/mTOR specific knockout mice (Fig. 4c), and the result was supported by the escalatory Eos-CFUs in allergic LysM^Cre^ Rheb^flox/flox^ mice (Fig. 4f). The consequences above indicated that mTOR myeloid specific ablation contributed to an elevated number of Eops in asthmatic mice, therefore leading to accelerated eosinophil development and enhanced levels of eosinophilic airway inflammation.

### The regulatory role of mTOR in eosinophil differentiation is mediated by Erk activation

**Figure 5:**
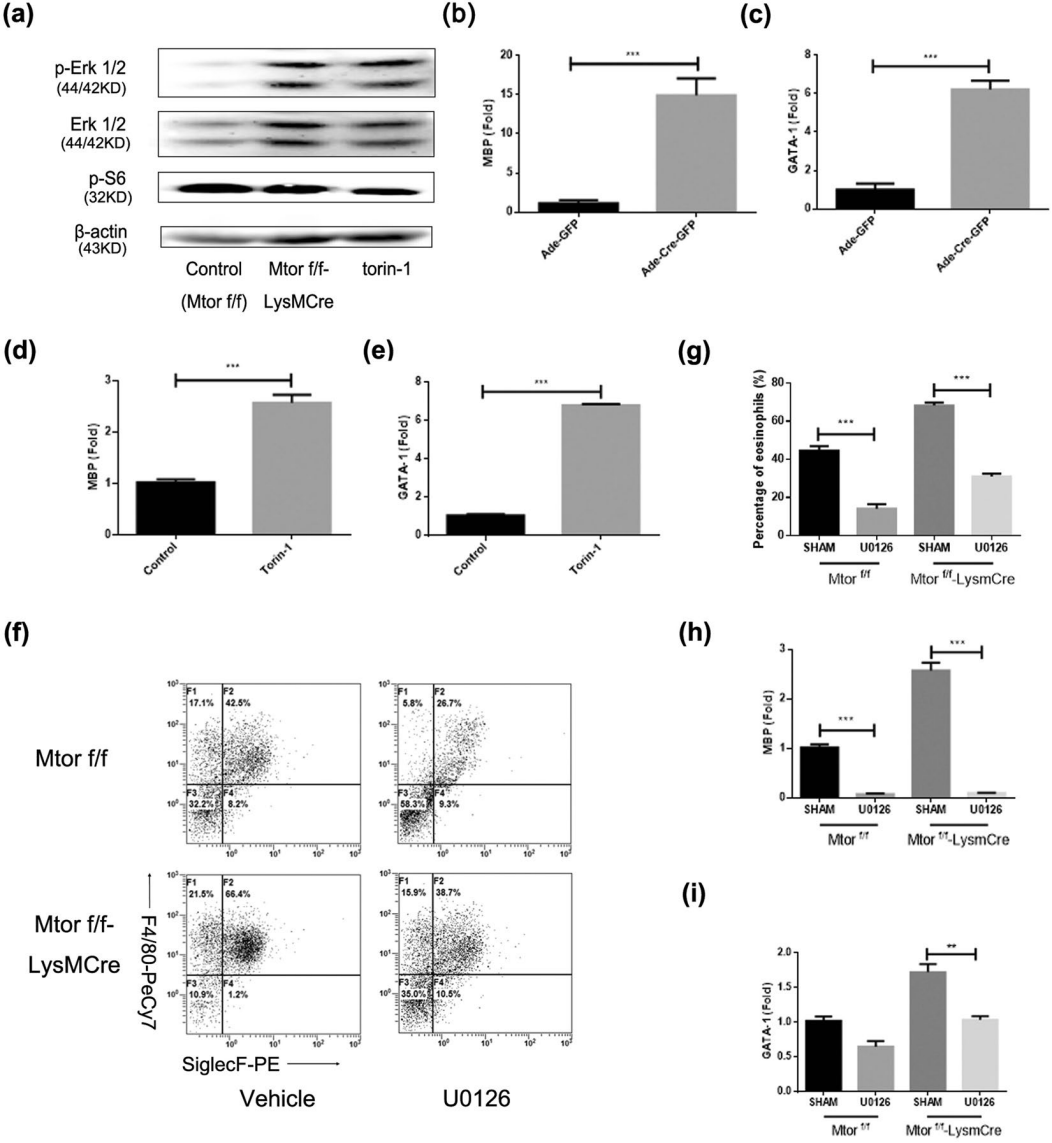
mTOR regulates eosinophil differentiation via p-Erk signalling. (**a**) Blots for p-Erk and Erk were displayed from the same gel. Phospho-Erk was firstly detected, then the antibody was eluted by 200 mM sodium hydroxide solution, and total Erk antibody incubation followed using the same membrane. Other blots were cropped by different gels.) Erk signal and p-Erk were detected using western blot on day 9. (**b**–**e**) On day 9, cells were harvested using Trizol reagent and mRNA performed inverse transcription. Quantitate PCR was performed to detect major basic protein (Mbp) and Gata-1 expression. Result B showed Mbp expression after adenoviral administration and result C displayed Gata-1 expression after adenoviral exposure. Result D revealed Mbp expression with torin-1 (250 nM) treatment and result E suggested Gata-1 expression after torin-1 exposure. (**f**,**g**) U0126 (20 μM) was used to inhibit phosphorylation of Erk, treatment of U0126 resulted in diminished eosinophil development in both strains (f). Results were analysed as means ± SEM (g). (**h**,**i**) Mbp (h) and Gata-1 (i) were also detected by Q-PCR post U0126 administration. Results were analysed as means ± SEM. All the results above were repeated by three independent experiments. *P < 0.05, **P < 0.01, ***P < 0.001.

With the purpose of exploring the possible molecular mechanism of eosinophil differentiation, BMDE was collected to perform western blot assays. Erk signalling was an imperative kinase in IL-5-induced eosinophilopoiesis^32^, and both p-Erk and Erk were detected by western blot assay (Fig. 5a). Phosphorylation of Erk1/2 was enhanced after mTOR specific deletion or by torin-1 treatment. Gata-1 is a decisive transcription factor in the maturation from GMPs to Eops, and previous studies have proven an enhanced expression of p-Erk accompanied with upregulated Gata-1 expression^27^. Quantitative real-time PCR analysis revealed that eosinophil-associated mRNA levels of eosinophil major basic protein (Mbp) and transcription factor Gata-1 were outstandingly augmented after adenoviral administration (Fig. 5b and c) or torin-1 treatment (Fig. 5d and e). Meanwhile, U0126, a specific Erk inhibitor, could invert the elevated percentage of eosinophils due to mTOR specific deletion (Fig. 5f and g). U0126 treatment also decreased Mbp amd Gata-1 expression (Fig. 5h and i). All the data together disclosed that mTOR obliteration may accelerate eosinophil differentiation by means of activating p-Erk, followed by the overexpressed transcription factor Gata-1.

### Myeloid specific impairment of mTOR deteriorates OVA-induced allergic airway inflammation in mice

**Figure 6:**
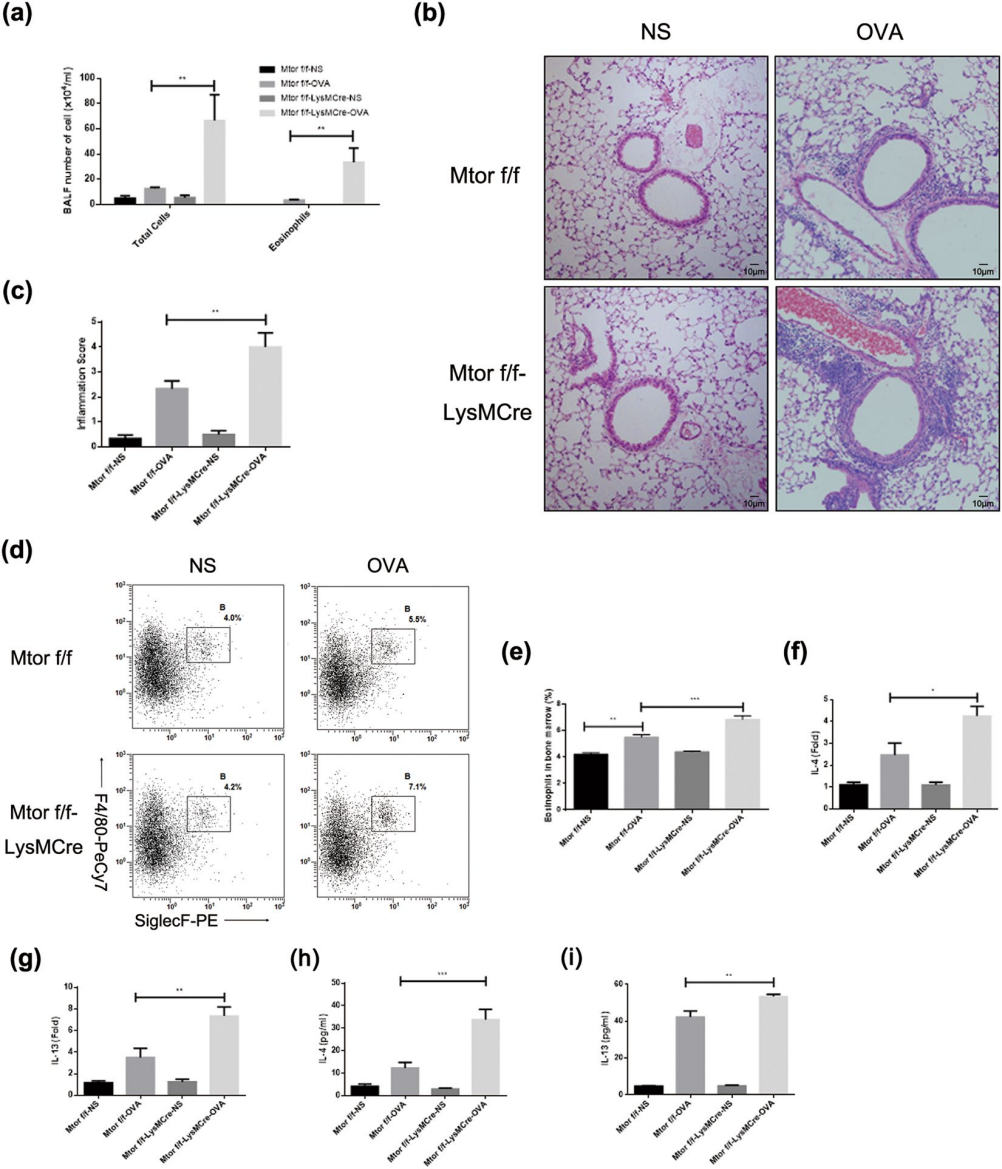
Myeloid knockout of mTOR deteriorates OVA-induced allergic airway inflammation. (**a**) Two strains of mice received ovalbumin (OVA) mixed with an injection of alum adjuvant on day 0 and day 14 for sensitization, then challenged with 1.5% OVA by nebulization. 24 hours after the last OVA exposure, mice were anaesthetized and sacrificed. Bronchoalveolar lavage fluids (BALFs) were obtained and total cells and eosinophils in BALFs were counted. (**b**,**c**) Representative histological images are shown (b) Inflammation scores were calculated according to H&E slides. (**d** and **e**) Bone marrow cells were also harvested and red blood cell lysis was performed. Eosinophil percentage was detected (d) and statistically analysed (**e**). (**f**–**i**) Th2 cytokines were detected by lung homogenates. F, mRNA expression of IL-4. G, mRNA expression of IL-13. H, protein concentration of IL-4. I, protein concentration of IL-13. Results were analysed as means ± SEM. All the results above were repeated by three independent experiments (n = 8 per group). *P < 0.05, **P < 0.01, ***P < 0.001.

Allergic asthma was characterized by eosinophil infiltration. Accordingly, accelerated eosinophilopoiesis may result in enhanced inflammatory levels in the airway. In an OVA-induced asthma model, mTOR myeloid selectively deletion mice showed an increased level of inflammatory cells in BALF, and so were eosinophils (Fig. 6a). The deteriorated levels of eosinophilic inflammation were further demonstrated by pathology evidence, followed by inflammatory scores (Fig. 6b and c) and immunohistochemistry staining of EPX (Fig. S5A and B). Meanwhile, we detected eosinophil percentages in the bone marrow of each group, and discovered that myeloid specific mTOR knockout LysM^Cre^ Mtor^flox/flox^ mice resulted in enhanced induction of eosinophils as SiglecF^+^F4/80^+^ cells (Fig. 6d and e). Moreover, Th2 immune response associated cytokines IL-4 and IL-13 were significantly increased in lung homogenates in LysM^Cre^ Mtor^flox/flox^ mice (Fig. 6f–i). To clarify the balance of lymphocytes, T cell subsets were also assessed. The balance of Th1/Th2 responses were disproportionate and reflected as impaired Th1 percentages and elevated Th2 proportions (Fig. S6A and B) in asthmatic knockout mice. The Th17 response and Treg percentage, however, appeared to remain the same level (Fig. S6C and D). All these data demonstrated that mTOR impairment in myeloid cells increased eosinophilia, which consequently resulted in elevated airway inflammation in response to allergen challenge.

### Myeloid Rheb selective deletion results in enhanced eosinophilic inflammation in airway

**Figure 7:**
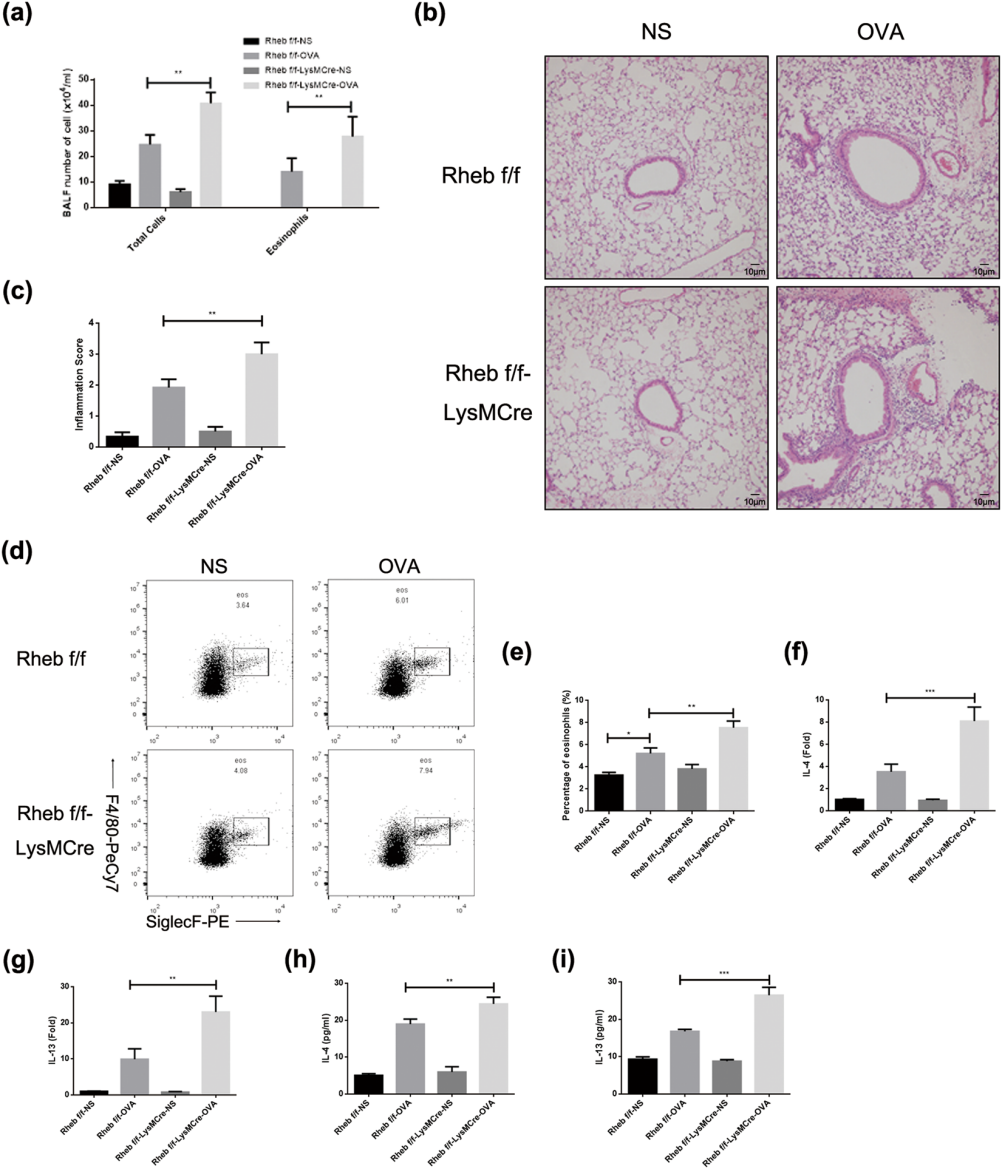
Myeloid knockout of Rheb deteriorates OVA-induced allergic airway inflammation. (**a**) OVA-induced allergic airway inflammation was established using two strains of mice as above. BALFs were obtained and total cells and eosinophils in BALFs were counted. (**b** and **c**) Representative histological images are shown (b). Inflammation scores were calculated according to H&E slides. (**d** and **e**) Bone marrow cells were also harvested and red blood cell lysis was performed. Eosinophil percentage was detected (d) and statistically analysed (e). (**f**–**i**) Th2 cytokines were detected by lung homogenates. f, mRNA expression of IL-4. g, mRNA expression of IL-13. h, protein concentration of IL-4. i, protein concentration of IL-13. Results were analysed as means ± SEM. All the results above were repeated by three independent experiments (n = 8 per group). *P < 0.05, **P < 0.01, ***P < 0.001.

Ras homolog enriched in the brain (Rheb) is a GTPase, and both mTOR and Rheb share a common pathway because the GTP-binding domain of Rheb unswervingly interacts with the mTOR complex, thereby kindling its kinase activity. Similarly, we discovered an increased level of airway inflammation and eosinophilic infiltration in BALF after Rheb specific knockdown in myeloid cells (Fig. 7a–c). In bone marrow, eosinophil percentage was also elevated in LysM^Cre^ Rheb^flox/flox^ mice after ovalbumin challenges (Fig. 7d and e). Similarly, the increased levels of IL-4 and IL-13 were detected in lung homogenates of LysM^Cre^ Rheb^flox/flox^ mice (Fig. 7f–i). These fallouts revealed that deletion of Rheb in myeloid cells also led to boosted eosinophilic airway inflammation.

## Discussion

**Figure 8:**
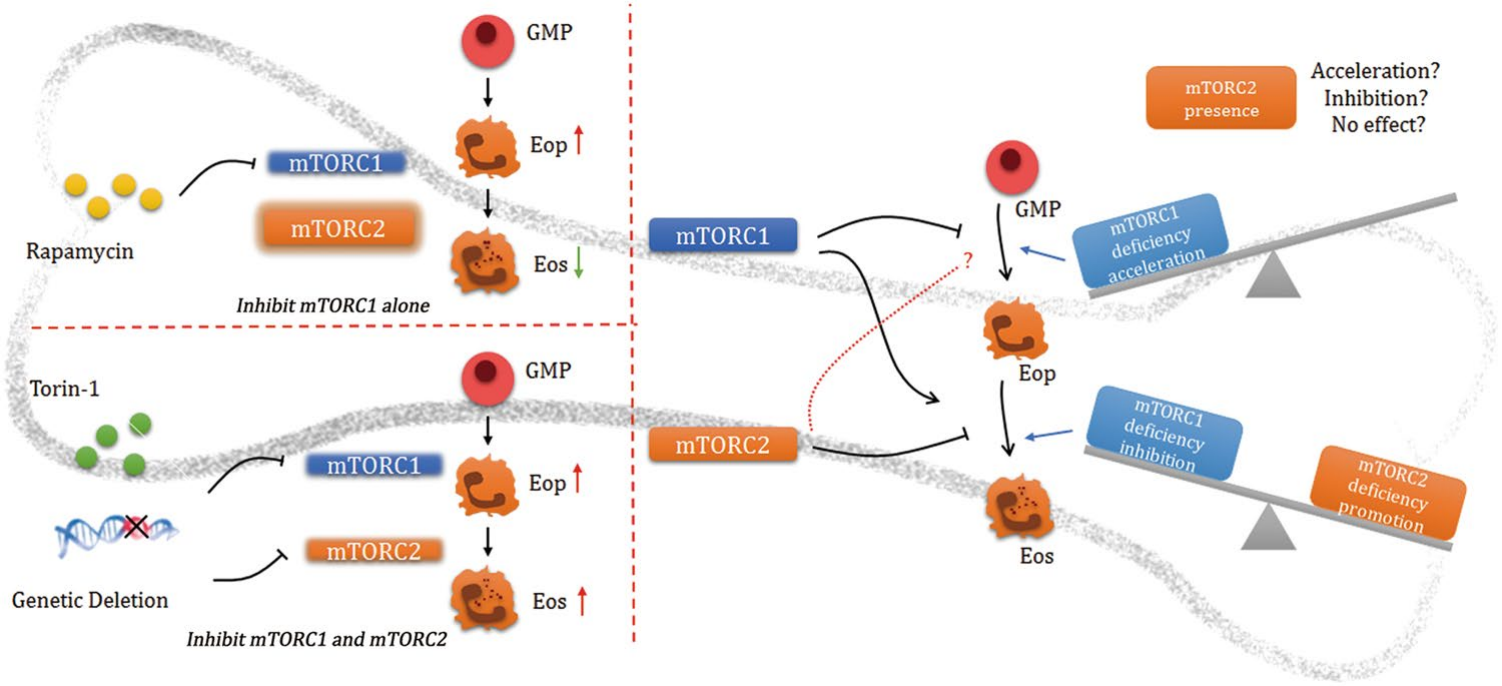
Summary. Administration of rapamycin results in increased Eop and diminished eosinophils by partially inhibits mTORC1, whereas torin-1 and genetic depletion of mTOR promotes both Eop and eosinophil development. We assumed that mTORC1 deficiency promotes Eop development, regardless of the effect mTORC2 exerts on Eop production from GMP. However, a loss of both mTORC1 and mTORC2 eventually increases the mature eosinophil, suggesting that mTORC2 plays a predominant role in suppressing eosinophil differentiation for Eop, regardless of, or overcoming the promoting effect of mTORC1 from Eop to mature eosinophil.

Eosinophil infiltration is regarded as a distinctive phenotype in asthmatic pathogenesis. Accordingly, the blockade of eosinophil differentiation may be a potential target in asthma therapy. Eosinophils originates from bone marrow derived hematopoietic stem cells and precursors^6,11^. However, few eosinophils exist in peripheral blood and lung tissue without allergen or parasite exposure. Once allergens are exposed, eosinophils can migrate to the blood stream and are then recruited to the pulmonary microenvironment under the control of IL-5 and eotaxins^6,8^. However, several studies focus on the dysfunction of airway epithelial cells or over-generated smooth muscles in asthma regardless of the regulation of eosinophil development. In our study, we demonstrated a key regulatory role of mTOR in eosinophil differentiation. Through the usage of experiments *in vitro* and *in vivo*, we discovered that the loss of mTOR contributed to elevated eosinophil differentiation both by conditional deletion or chemical inhibition. Meanwhile, mTOR myeloid specific knockout mice displayed deteriorated airway inflammatory responses after ovalbumin exposure, with an increased number of Eops (Fig. 8). To investigate haematopoietic cell differentiation, it was common to use Vav1^Cre^ mice to resect specified gene segments in an early stage of development^28,33^. However, we could not obtain live Vav1^Cre^ Mtor^flox/flox^ mice (data not shown). As mTOR is essential in HSCs function^16^, we assumed that the deletion of mTOR at an early stage of haematopoiesis could lead to embryo mortality. It was noteworthy that mTOR deficiency in myeloid cells led to increased Eops, and may be the reason why airway inflammatory penetration was exacerbated in the absence of mTOR. Consequently, mTOR activation was indispensable in prohibiting excessive eosinophil development, which may be a potential therapeutic target in asthmatic treatment.

To the best of our knowledge, there are no eosinophil-lineage specific knockout mice at present. Therefore, we utilized an LysM^Cre^ system instead. The LysM^Cre^ system involves other myeloid cells, including neutrophils, monocytes, macrophages, and dendritic cells^34^, and we could not dismiss the possible effects of those cells in allergic mice models *in vivo*. Nonetheless, we have demonstrated at least that mTOR activity (displayed as p-S6) was impaired in eosinophils from LysM^Cre^ Mtor^flox/flox^ mice (Fig. S7), revealing that mTOR complexes were involved in eosinophil development *in vivo*. Also, the *in vitro* experiments further demonstrated that mTOR indeed modulated eosinophil differentiation.

Ras homolog enriched in the brain (Rheb) lies upstream of mTOR, and acts as a GTPase that can bind to the GTP-binding domain of mTOR and directly interact with it, thereby altering its kinase activity^15^. Hence, Rheb changes may represent mTOR alternation under most circumstances. With the basis of this theory, LysM^Cre^ Rheb^flox/flox^ mice were established to verify previous data collected from mTOR disruption. Since LysM^Cre^ also participated in neutrophil differentiation and monocyte/macrophage maturation^34^, influences of mTOR in these two types of inflammatory cells were also observed. Interestingly, we dicovered that mTOR myeloid deletion promoted eosinophil lineage colony formation, but restrained G-CSF-induced neutrophilic development. Meanwhile, M-CSF-mediated macrophage differentiation reflected as being equivalent after mTOR knockdown. However, the detailed mechanisms mediating the differential functions of mTOR in granulocyte development remain unclear, and further research ought to be conducted.

mTOR interacts with various proteins to form two components, mTORC1 and mTORC2. mTORC1 is regarded as a vital modulator in protein synthesis, lipid manufacture, autophagy, cell survival status^14,15^, and so on. mTORC1 is required for maintaining the function of hematopoietic stem cells, and mice receiving Raptor-loss bone marrow showed as having a poor survival proportion post bone marrow transcription^16^. Rapamycin inactivates mTORC1 through binding the FK506-binding protein FKBP12, and can directly interact with mTORC1 and inhibits its activity^35–37^. It is reported that rapamycin treatment could alleviate allergic asthma in mice via hindering eosinophilopoiesis, correspondingly blocked the formation of airway hyperresponsiveness and the secretion of IgE^22,23^. Interestingly, rapamycin cannot always act as a protective factor in asthma^24^. Similarly, the role of rapamycin in insulin resistance caused by inflammatory stress regulation was also enigmatic^38^.

It might be of interest to note that exogenous rapamycin administration may result in inhibited eosinophil differentiation^23^. However, in our current investigation, accelerated eosinophil development induced by mTOR conditional impairment or torin-1 inhibition has been demonstrated. Rapamycin is characterized as a canonical inhibitor of mTORC1, but mTORC2 cannot be affected by acute rapamycin administration. Mounting evidence has suggested that acute exposure of rapamycin can inhibit mTORC1, but prolonged administration can hinder the assembly of mTORC2 via the Akt axis^39,40^. It is also noteworthy that rapamycin restrained eosinophil differentiation, but with an increased number of Eops^23^, thereby implying a possible maturation dysfunction in eosinophil development by rapamycin. In our research, mTOR deletion, or torin-1 administration, also resulted in increased numbers of Eops and the number of elevated mature eosinophils. A plausible explanation to describe this dissimilarity is that mTORC2 may also be an important modulator in eosinophil development, and that mTORC1 and mTORC2 exert differential effects. As summarized in Fig. 8, mTORC1 may selectively inhibit myeloid precursor (GMP) to differentiate toward eosinophil lineage (Eop), while promotes Eop to differentiate into eosinophils. Therefore, in the case of rapamycin treatment, the loss of mTORC1 activity resulted in increased Eop accumulation, but decreased mature eosinophils. However, mTORC2 may display opposite effects to mTORC1, at least in the differentiation stage from Eop to eosinophils. In the case of torin-1 or mTOR genetic deletion, the activity of both mTORC1 and mTORC2 are suppressed. In these cases, increased Eop suggests that mTORC1 deficiency plays a predominant role in promoting Eop development, regardless of whether mTORC2 deficiency would promote, inhibit, or exert no effect on Eop production from GMP. On the contrary, from Eop to mature eosinophil, loss of both mTORC1 and mTORC2 eventually increases mature eosinophil, suggesting that mTORC2 plays a predominant role in suppressing eosinophil differentiation for Eop, regardless of, or overcoming the promoting effect of mTORC1 in this stage. Nonetheless, a more specific deletion of mTORC1 or mTORC2, including Raptor or Rictor conditional deficiency, would be more appreciable to demonstrate the exact functions of these two mTOR complexes in eosinophil development, which might be warranted in future studies.

Likewise, recent studies have shown the contradictory effects of two complexes of mTOR in haematopoiesis-related researches. In CD8^+^ T cells generation, it was stated that mTORC1 mainly affected effector responses of CD8^+^ T cells, while mTORC2 selectively influenced memory regulation^18^. Meanwhile, macrophage polarization was regulated contrarily by two components of mTOR. Depletion of mTOR using rapamycin contributed to M1 polarization, while secreting increased IL-12 and decreased IL-10 after stimulating with LPS^19,41^. RAD001 was an analogue of rapamycin, and is also an mTOR inhibitor applied in Han’s research^42^. In Han’s work, regardless of treatment prevention, the therapeutic administration of RAD001 would promote M2 macrophage polarization at the peak phase of experimental autoimmune neuritis rat models. Hence, two distinct parts of mTOR complexes may have the potential to display diverse regulatory roles in cellular processes and in disease pathogenesis.

We attempted to search the paradoxical alternation of the activities of each complex (Fig. S8), and the dominant discrepancy was the p-erk signal. It is widely accepted that p38-MAP kinases-Erk1/2 axis exhibits great influence on eosinophil development, degranulation, and cytokine secretion^27,32,43,44^. Activation of Erk1/2 also plays a vital role in Siglec-8 mediated pro-apoptotic signals in activated eosinophils^45^. Therefore, administration of the Erk1/2 inhibitor U0126 contributed to eosinophil differentiation dysfunction. Notably, numerous research highlighted a crosstalk of Ras-Erk signalling and PI3K-mTOR pathways^46,47^, and the loss of mTOR mostly reflected as the augmented expression of p-Erk in several types of cells^48–50^. In our data, we first explored phosphorylation of Erk1/2 using bone marrow-derived eosinophils, and discovered that p-Erk1/2 was enhanced in BMDE originated from LysM^Cre^ Mtor^flox/flox^ mice. Accordingly, we assumed that mTOR may limit eosinophil differentiation via hindering phosphorylation of Erk1/2. To test our hypothesis, U0126, which is a pharmacological inhibitor of Erk1/2, was accompanied with the IL-5-induced differentiation process. Our results indicated that U0126 could not only hinder eosinophil development from NAMNCs, but eliminate mTOR selective ablation, which induced excessive eosinophil differentiation.

Gata-1 is considered essential in the development from myeloid progenitors to eosinophil lineage-committed progenitors, and the expression of eosinophil-associated genes^9,10,28^. Deletion of high-affinity double-palindromic GATA sites in murine Gata-1 promoters contributed to the deficiency of eosinophil lineage^51^. We detected the modification of Gata-1 expression after knocking down mTOR and p-Erk1/2. Impairment of mTOR by adenoviral Cre recombinase administration or pharmacological inhibition led to overexpressed Gata-1, which implied a potential accelerated eosinophil differentiation. Moreover, BMDE treated with U0126 revealed decreased percentages of eosinophils together with a reduced Gata-1 expression. The above data revealed a hypothesis that mTOR ablation at least partially promoted eosinophil differentiation based on upregulating Gata-1 expression via enhanced phosphorylation level of Erk1/2 signal.

Collectively, our data uncovered the differential effects of mTOR in regulation of eosinophil development, which was likely due to the distinct functions of the mTOR complex 1 or 2; thus, exerting a pivotal implication in eosinophil-associated diseases.

## Electronic supplementary material

Supplement Figures
