# Supplementary material for: mTOR complexes differentially orchestrates eosinophil development in allergy
Source: Sci Rep. 2018 May 2;8:6883. doi: 10.1038/s41598-018-25358-z (PMC5932055; doi:10.1038/s41598-018-25358-z)
Supplement: Supplementary file 1 — Supplement Figures [file 41598_2018_25358_MOESM1_ESM.pdf]

mTOR complexes differentially orchestrates eosinophil development in allergy

Running title: mTOR orchestrates eosinophil development

Chen Zhu1, Lixia Xia1, Fei Li1, Lingren Zhou1, Qingyu Weng1, Zhouyang Li1, Yinfang Wu1, Yuanyuan Mao2, Chao Zhang1, Yanping Wu1, Miao Li1, Songmin Ying1,3, Zhihua Chen1, Huahao Shen1, Wen Li1

1 Department of Respiratory and Critical Care Medicine, The Second Affiliated Hospital, Zhejiang University School of Medicine, Hangzhou, Zhejiang 310009, China.

2 Department of Respiratory Medicine, Ningbo No.2 Hospital, Ningbo, Zhejiang 315010, China.

3 Department of Pharmacology, Zhejiang University School of Medicine, Hangzhou, Zhejiang 310058, China.

Correspondence should be addressed to Wen Li (liwen@zju.edu.cn)

Supplementary figure legends

Figure S1 Deletion of mTOR displays differential regulation in G-CSF-induced or M-CSF-induced colony forming. Scheme of colony forming was described previously, but G-SCF and M-SCF was mixed with cell suspension before seeded instead of IL-5. Adenoviral Cre recombinase administration remarkably reduced the quantity of G-CSF-induced CFUs (A) but did not alter the amount of CFUs induced M-CSF (B). Results were analysed as means ± SEM, statistically analysed by Student's T test. Each results were triplicated by three individual

experiments. ** P<0.01, n.s. not significant.

Figure S2 No significance of WBC and eosinophil was shown in peripheral blood in mTOR knockout mice. Peripheral blood was collected during sacrifices of allergic mice, and mixed with heparin in case of coagulation. Total WBC cells (A), percentage (B) and number (C) of eosinophils in blood samples were detected. Results were analysed as means ± SEM and triplicated. n.s. not significant.

Figure S3 Apoptotic level was not altered after mTOR myeloid specific knockdown with IL-5 transgenic mice. NJ1638 was a mouse strain whose $CD3^{+}$ cells was received an IL-5 encoding fragment insertion, subsequently displayed as eosinophil overload in peripheral blood and bone marrow. $LysM^{Cre}$ Mtor $^{flox/flox}$ mice cross-bred with NJ1638 mice to obtain abundant eosinophils. (A) Eosinophil percentage was detected in bone marrow of IL-5-transgenic $LysM^{Cre}$ Mtor $^{flox/flox}$ mice. (B) Bone-marrow-derived cells were culture in IMDM medium mixed with 10% heat-inactivated serum for 3h, 6h and 12h. Representative dot plots were shown. (C-D) Apoptosis was detected in 3h, 6h and 12h and analysed in total cells (C) and eosinophils (D). Results were analysed as means ± SEM and triplicated. n.s. not significant.

Figure S4 Early-stage progenitors were not altered in myeloid specific depletion mice. In flow cytometric analysis of Eops, earlier stage of hematopoietic precursors were also detected. A, LSKs number in per 10^{6} cells of allergic Mtor knockout mice. B, CMPs number in per 10^{6} cells of allergic Mtor knockout mice. C, GMPs number in per 10^{6} cells of allergic Mtor knockout mice. D,

LSKs number in per 10^6 cells of allergic Rheb knockout mice. E, CMPs number in per 10^6 cells of allergic Rheb knockout mice. F, GMPs number in per 10^6 cells of allergic Rheb knockout mice. Results were analysed as means ± SEM and triplicated (n=5 per group). n.s. not significant.

Figure S5 Growing eosinophil infiltration was detected in asthmatic mTOR knockout mice. Epx antibody applied for immunohistochemistry was a generous gift from Prof. James J. Lee. Epx^+ eosinophils accumulated around asthmatic airway as represented as (A), mTOR depletion depraved the progression (B).

Figure S6 Th2 response was enhanced in myeloid specific deletion of mTOR after OVA exposure. The lung tissue was digested into single cell suspension then stained for the detection of Th1, Th2, Th17 and Treg cellularity. The Th1 response was inhibited in mTOR deletion mice following OVA exposure (A), meanwhile the Th2 percentage augmented (B). Nonetheless, Th17 (C) and Treg (D) proportion appeared to demonstrate invariable.

Figure S7 mTOR was deleted by $LysM^{Cre}$ system in eosinophils. Eosinophils was purified by percoll (GE healthcare) isolation from peripheral blood of IL-5-transgenic $LysM^{Cre}$ Mtor $^{flox/flox}$ mice, the purity of eosinophil (marked as $SiglecF^+F4/80^+$) was >95% (A). The expression of mTOR, which displayed as p-S6 as previously described, was indeed knockdown in $LysM^{Cre}$ Mtor $^{flox/flox}$ mice compared with that of control mice (B). Furthermore, expression of p-S6 was also detected by western blot assay (C).

Figure S8 Paradoxical alternation in p-Erk signal post various mTOR prohibition. Main

downstream signal proteins of each complex of mTOR were detected by western blot. The expression of p-mTOR, mTOR, p-Akt, p-S6 and p-PKCα were consistently diminished after various treatment. However, p-Erk signal displayed conflicting decline in rapamycin administration compared to other treatment.

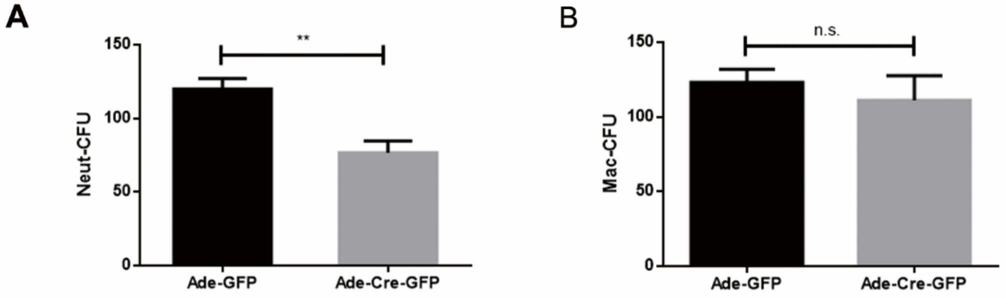

Figure S1 Deletion of mTOR displays differential regulation in G-CSF-induced or M-CSF-induced colony forming.

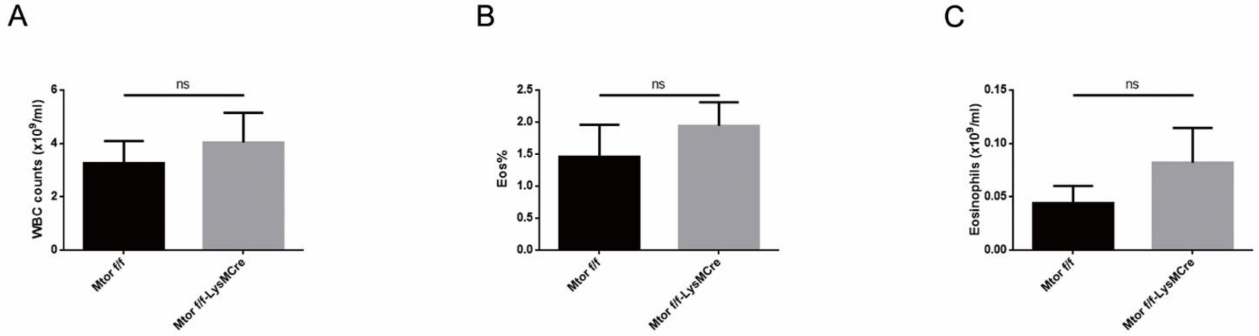

Figure S2 No significance of WBC and eosinophil was shown in peripheral blood in mTOR knockout mice.

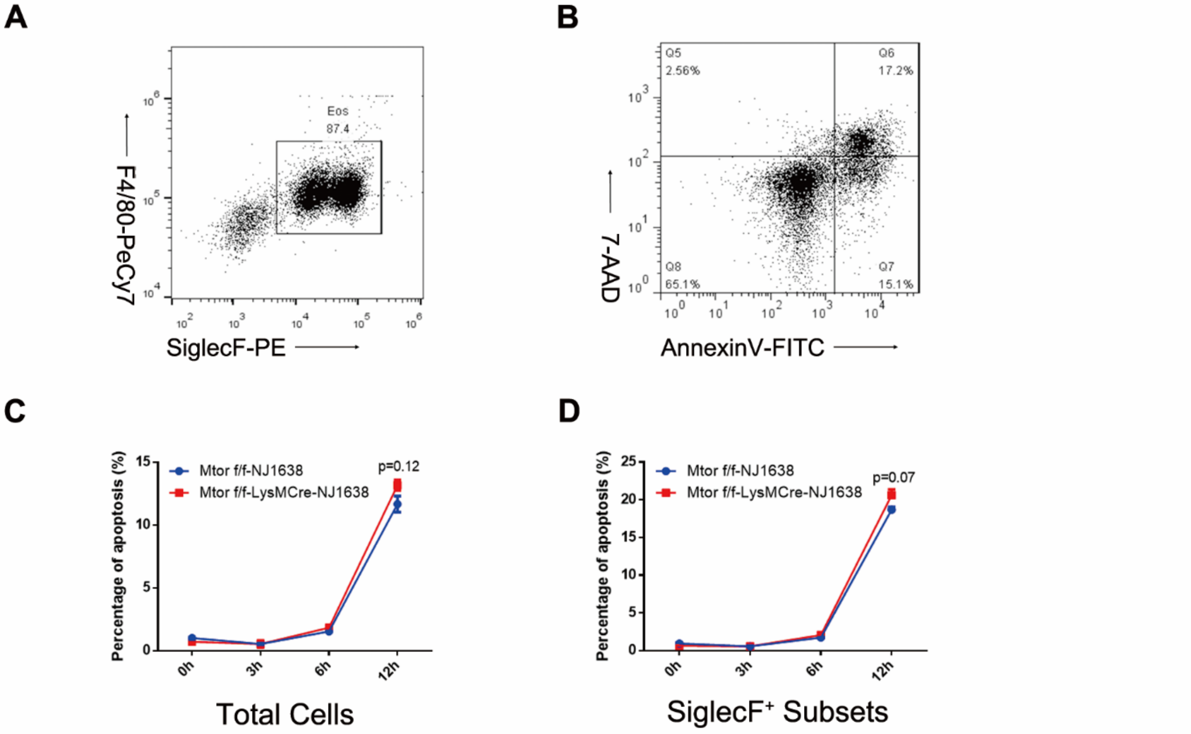

Figure S3 Apoptotic level was not altered after mTOR myeloid specific knockdown with IL-5 transgenic mice.

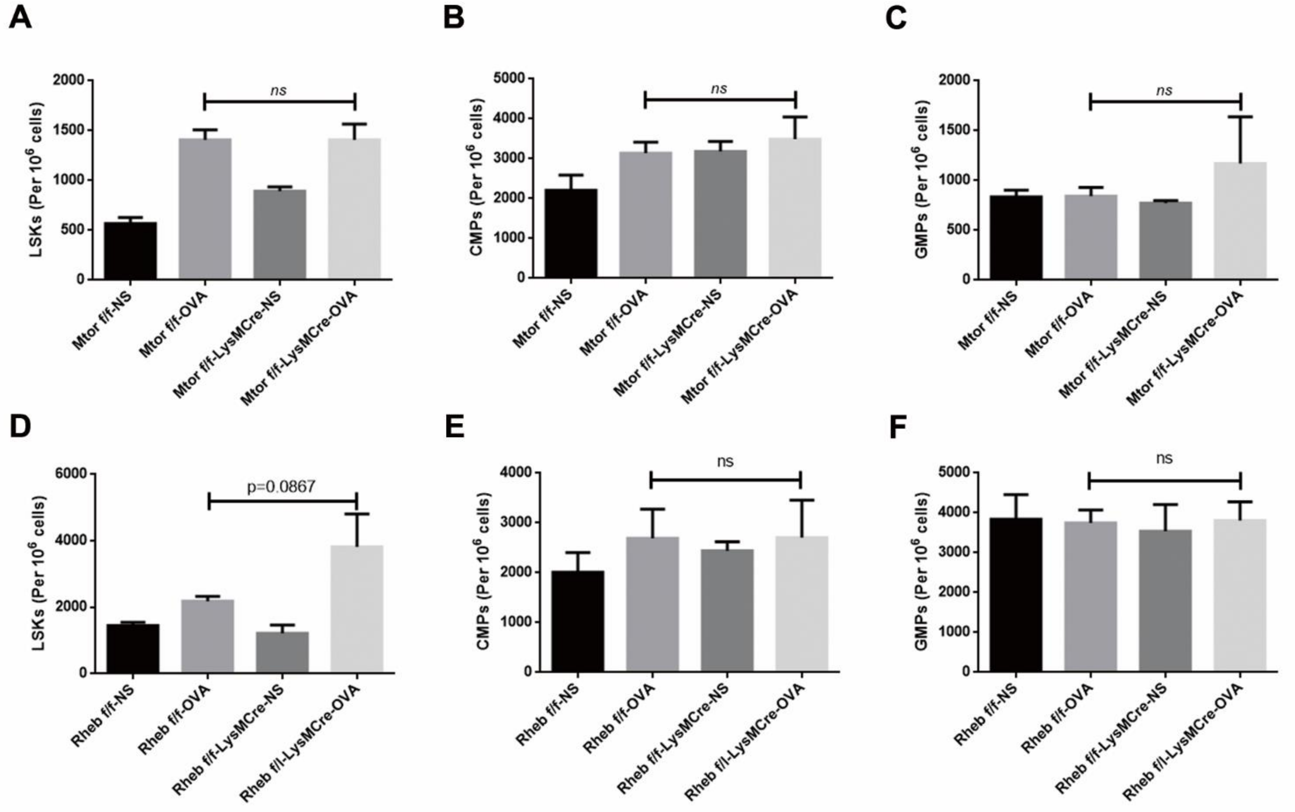

Figure S4 Early-stage progenitors were not altered in myeloid specific depletion mice.

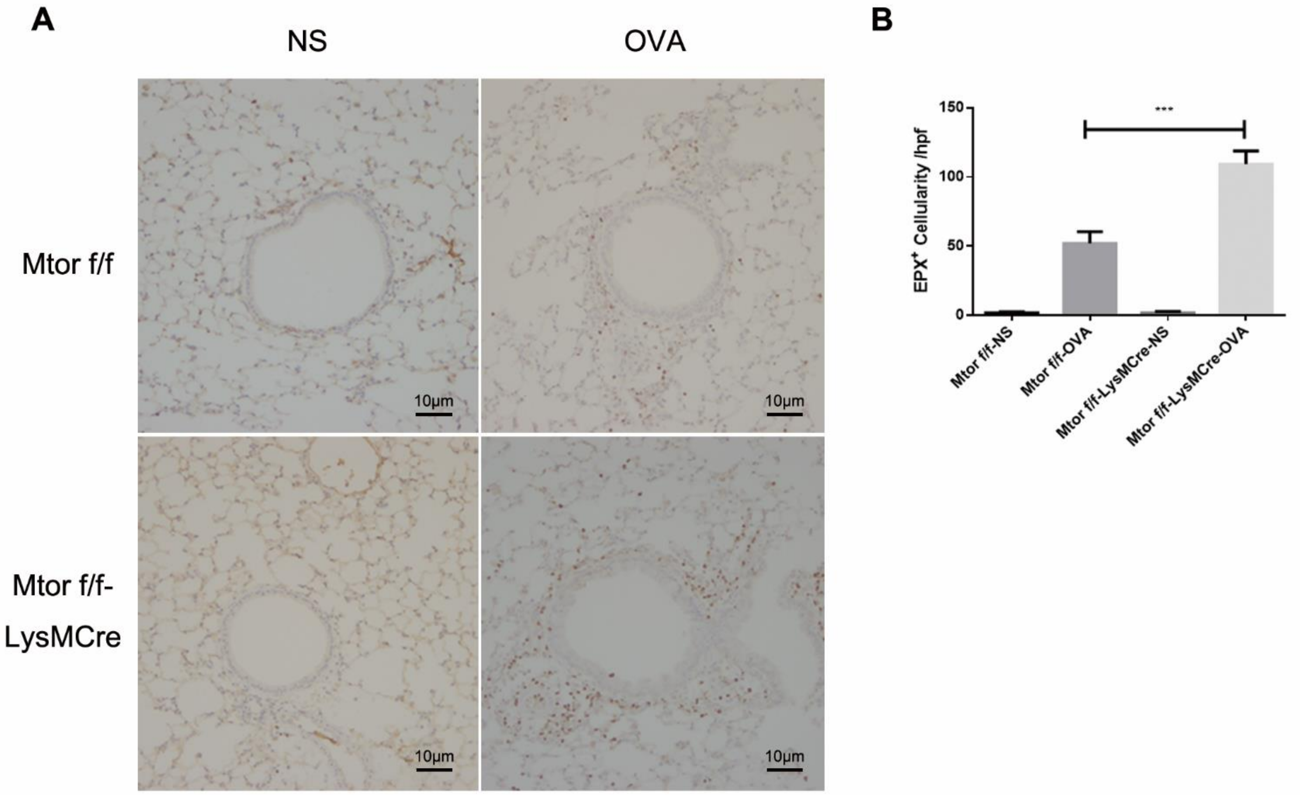

Figure S5 Growing eosinophil infiltration was detected in asthmatic mTOR knockout mice.

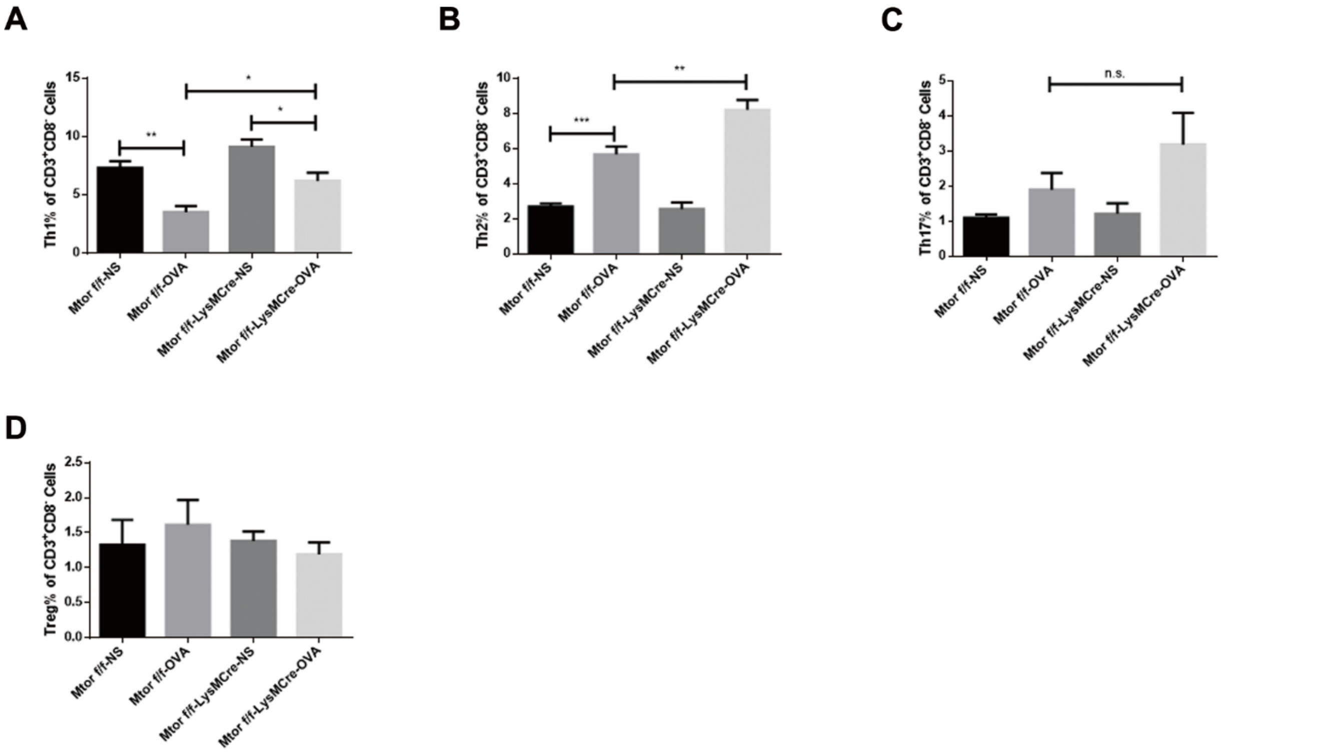

Figure S6 Th2 response was enhanced in myeloid specific deletion of mTOR after OVA exposure.

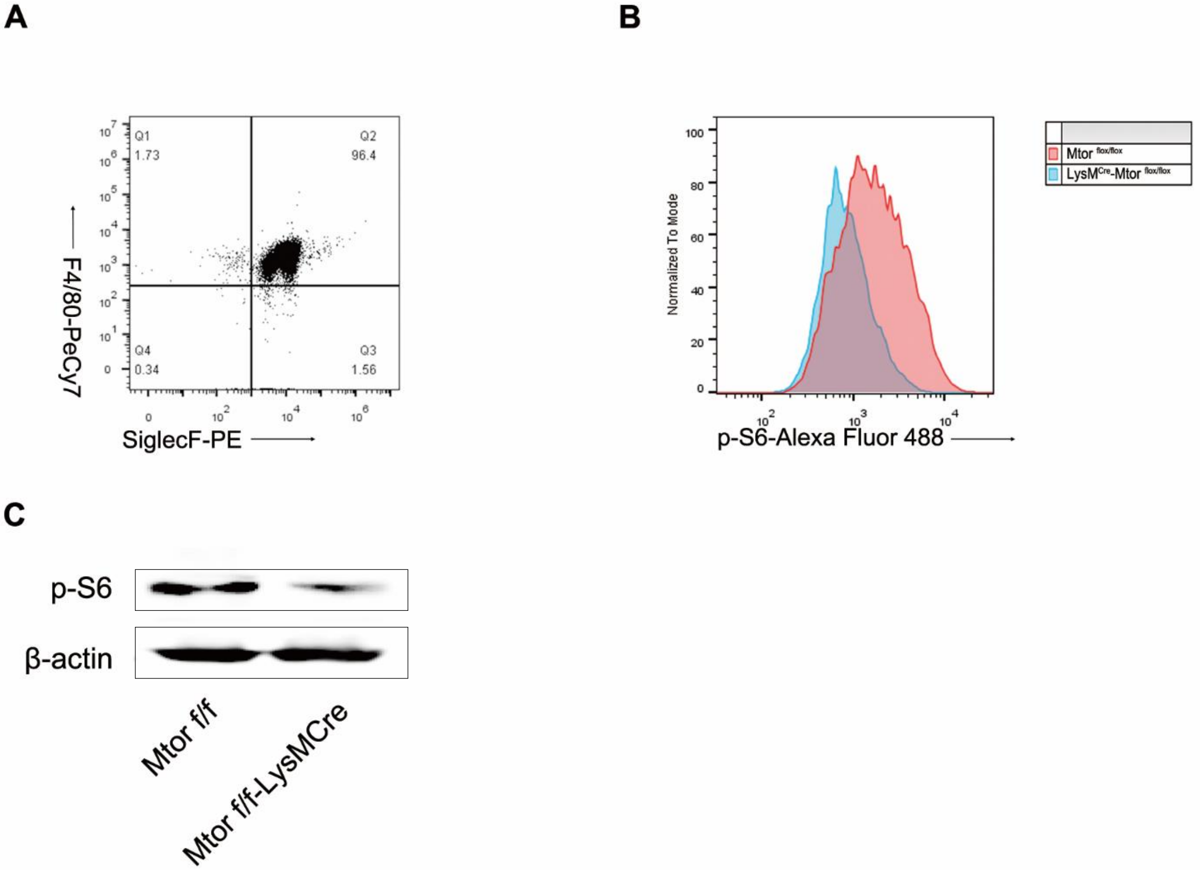

Figure S7 mTOR was deleted by LysM^{Cre} system in eosinophils.

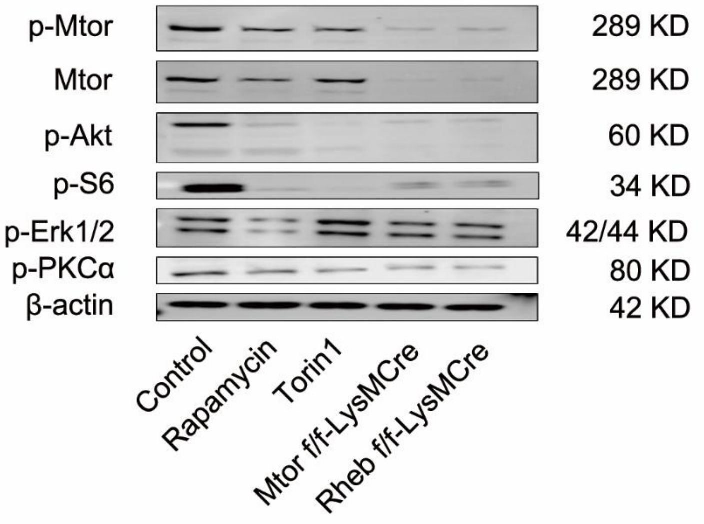

Figure S8 Paradoxical alternation in p-Erk signal post various mTOR prohibition.

Figure 1C

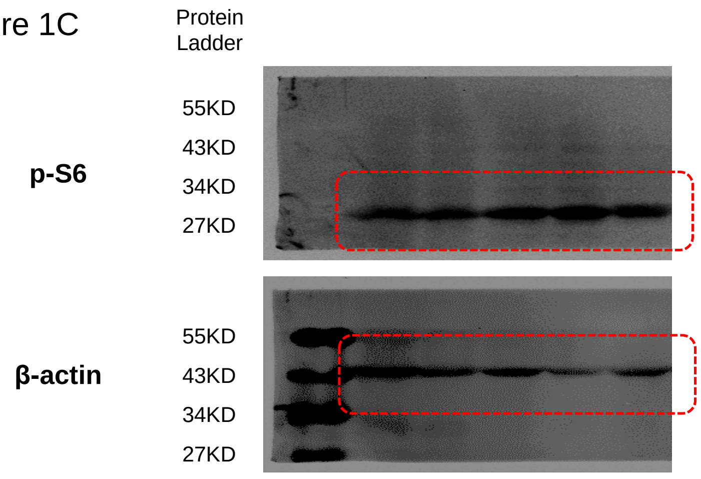

Figure 1J

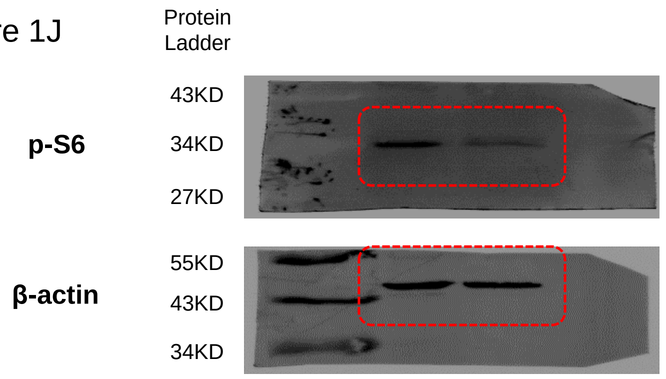

Figure 5A

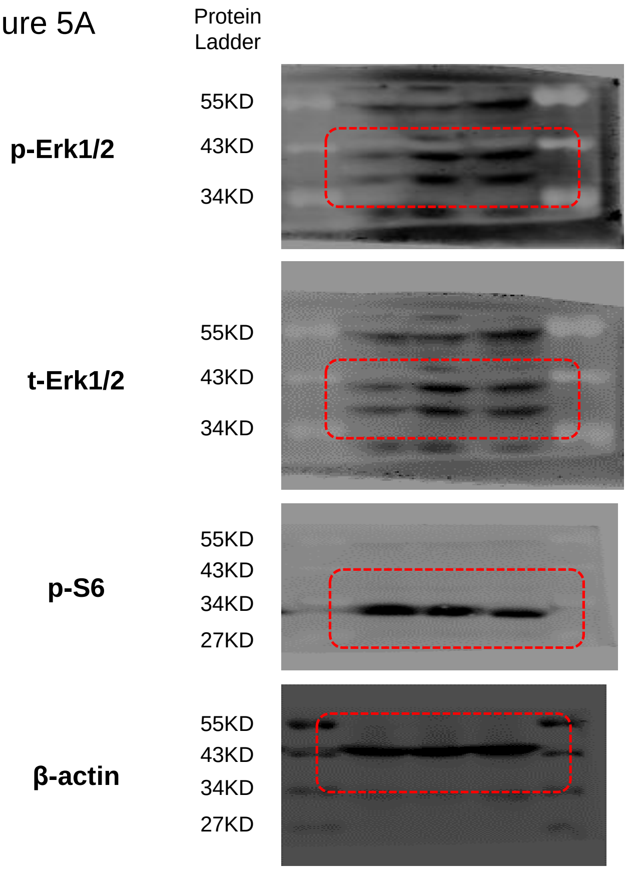

Supplementary Figure Full-length blot images

Figure S7C

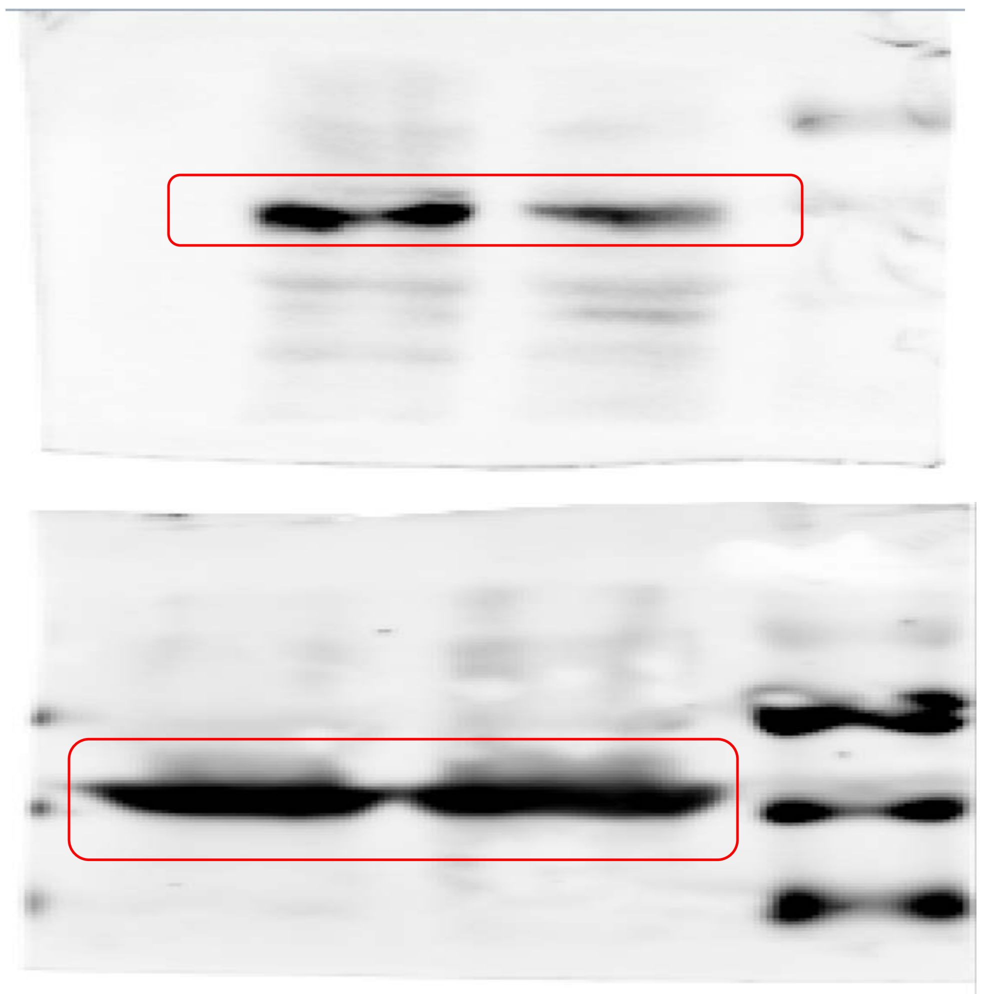

Figure S8

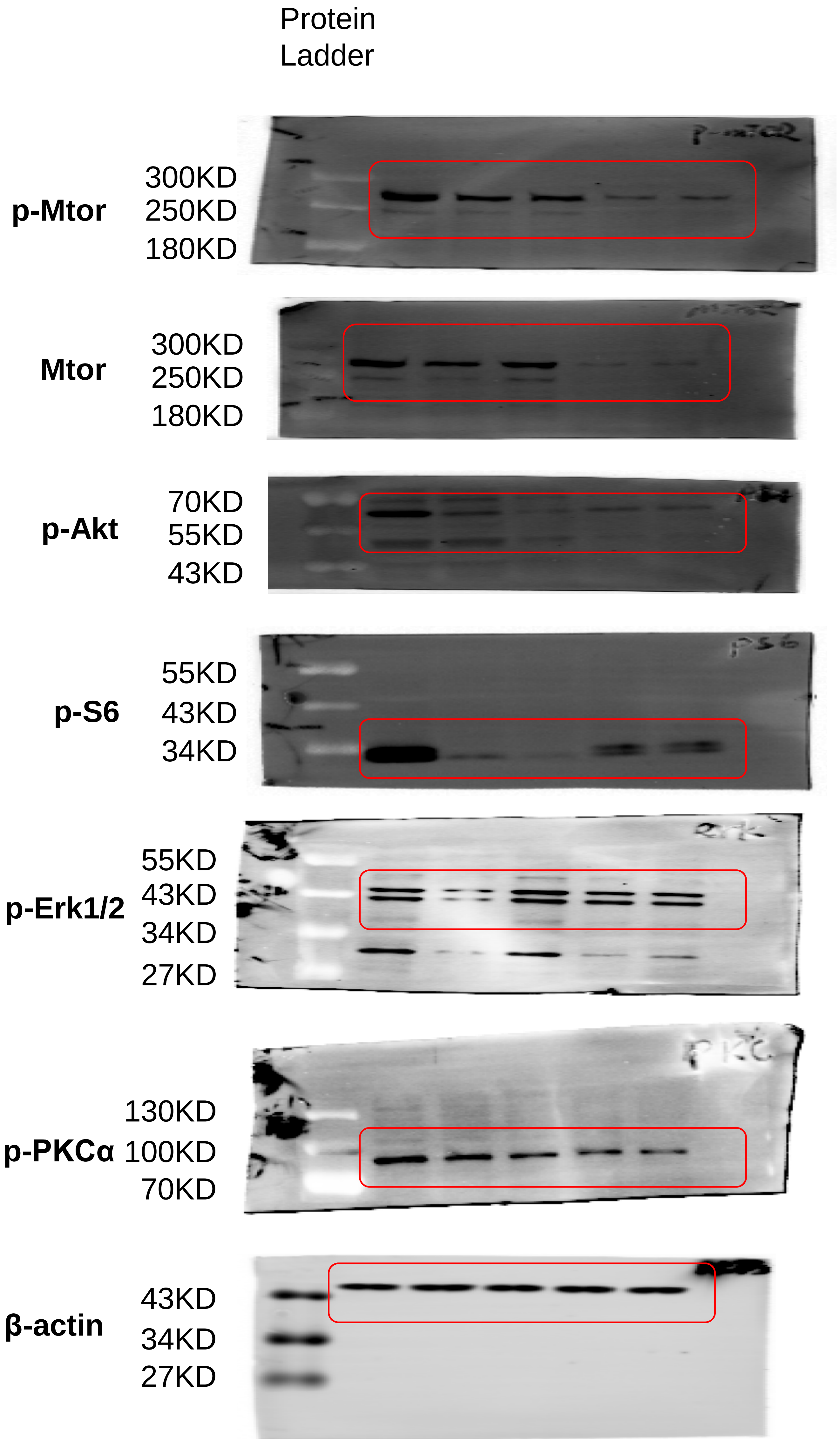
Protein Ladder
p-Mtor
300KD
250KD
180KD
Mtor
300KD
250KD
180KD
p-Akt
70KD
55KD
43KD
p-S6
55KD
43KD
34KD
p-Erk1/2
55KD
43KD
34KD
27KD
p-PKCα
130KD
100KD
70KD
β-actin
43KD
34KD
27KD
